# Temporal Discordance of the Inflammation, Catabolism and Immunosuppression Triad in Chronic Critical Illness

**DOI:** 10.3390/medicina62091686

**Published:** 2026-09-03

**Authors:** Valery Likhvantsev, Levan Berikashvili, Petr Polyakov, Mikhail Yadgarov, Artem Kuzovlev, Alexey Yakovlev, Andrey Grechko

**Affiliations:** 1 Department of Clinical Trials, Federal Research and Clinical Center of Intensive Care Medicine and Rehabilitology, 107031 Moscow, Russia; lberikashvili@fnkcrr.ru (L.B.); p.polyakov@fnkcrr.ru (P.P.); myadgarov@fnkcrr.ru (M.Y.); artem_kuzovlev@fnkcrr.ru (A.K.); ayakovlev@fnkcrr.ru (A.Y.); fnkcrr@fnkcrr.ru (A.G.); 2 Department of Anesthesiology and Intensive Care, I.M. Sechenov First Moscow State Medical University of the Russian Ministry of Health (Sechenov University), 119048 Moscow, Russia

**Keywords:** chronic critical illness, inflammation, catabolism, immunosuppression, PICS, temporal discordance, intensive care unit

## Abstract

*Background and Objectives*: Chronic critical illness (CCI) is a growing problem in modern intensive care, though its pathophysiology remains incompletely defined. While the ICS triad is thought to perpetuate organ dysfunction, the temporal interplay between its components has not been studied. The objective was to characterize the temporal dynamics of each component and to assess their synchrony in CCI patients. *Materials and Methods*: This retrospective cohort study analyzed data from the Russian Intensive Care Dataset (RICD v3.0). Adult ICU patients with at least one documented ICS episode (CRP > 20 mg/L, albumin < 30 g/L, lymphocytes < 0.8 × 10^9^/L within 24 h) were included. Daily binary statuses were generated for each domain using an episode-persistence algorithm. Pairwise concordance was assessed using Cohen’s κ with bootstrap resampling and generalized estimating equation (GEE) models; domain burden, episode frequency, and status combinations were also evaluated. *Results*: A total of 820 CCI patients contributed 18,525 non-critical patient-days. CRP and albumin domains were persistently positive (median burden 100% both), while lymphocyte positivity was brief (median burden 34.2%) and recurrent (≥2 episodes in 45.1%). CRP and lymphocyte domains were discordant in 81.2% of days (κ = −0.024; OR 0.60; Holm-adjusted *p* = 0.005); lymphocyte and albumin domains in 77.0% (κ = −0.021; OR 0.49; Holm-adjusted *p* < 0.001). CRP and albumin were largely concordant (78.5%; κ = 0.299; OR 2.71; Holm-adjusted *p* < 0.001). Isolated lymphopenia was rare (0.6%), whereas complete compensation was seen in 7.7% of observations. *Conclusions*: These findings suggest that the ICS triad components follow distinct, temporally discordant trajectories after CCI onset, questioning the view of CCI as a unified state. While periods of complete compensation were observed, their relationship to clinical resolution of CCI requires further investigation.

## 1. Introduction

Chronic critical illness (CCI) has emerged as a major challenge in modern intensive care [1,2,3]. Advances in resuscitation and organ support have reduced early mortality, paradoxically giving rise to a growing population of patients who survive the acute phase but remain dependent on intensive care for weeks or months [4,5,6,7,8,9]. Epidemiological data indicate that CCI develops in approximately 7–8% of ICU admissions, with in-hospital mortality approaching 30% and one-year mortality exceeding 45% [10,11].

Despite its clinical and prognostic significance, the underlying pathophysiological drivers of CCI remain incompletely understood. A growing body of evidence points to a triad of persistent inflammation, immunosuppression, and catabolism, first described as (PICS) by Gentile and colleagues [12], and later reframed as ICS when the temporal component is not emphasized [13], as a key mechanism perpetuating organ dysfunction and poor outcomes [14,15]. Conceptually, CCI denotes the clinical state of prolonged intensive care dependence, while PICS and ICS refer to the underlying pathophysiological processes. It is important to distinguish between PICS and the ICS triad. PICS (persistent inflammation, immunosuppression, and catabolism syndrome) incorporates a temporal dimension, typically defined by the persistence of these abnormalities beyond 14 days of critical illness. In contrast, the ICS triad refers specifically to the concurrent presence of the three derangements (inflammation, catabolism, and immunosuppression) without an inherent time requirement. Thus, ICS describes the pathophysiological state itself, regardless of when it occurs or how long it persists. This triad is hypothesized to constitute a self-sustaining vicious cycle in which inflammation drives catabolism and suppresses immune function, while immunosuppression increases susceptibility to secondary infections that in turn fuel further inflammation [12,16]. The prognostic relevance of this triad has been well established: its presence identifies patients at substantially increased risk of death and disability, regardless of the duration of ICU stay [15,17,18].

Nevertheless, the temporal architecture of this triad has remained surprisingly underexplored. The field has largely conceptualized ICS as a relatively stable state that, once established, persists until the patient either recovers or dies [12,19]. Yet clinical experience and longitudinal laboratory data suggest that the individual components of the triad may not move in lockstep. CRP, albumin, and lymphocyte count often fluctuate independently, raising the possibility that the triad is not a fixed condition but a dynamic one, with periods of convergence, when all three components are abnormal, alternating with periods of divergence, when at least one component is transiently compensated. This concept of temporal discordance, the independent time-varying behavior of the inflammatory, catabolic, and immunosuppressive components, has been proposed but never systematically examined [20].

To address this gap, we designed a longitudinal, patient-day-level analysis of biomarker domains in a large cohort of patients with chronic critical illness. The objective was to characterize the temporal behavior of each component of the triad and to quantify the degree of synchrony between components using pairwise concordance analysis.

## 2. Materials and Methods

### 2.1. Source of Data

Data were obtained from version 3.0 of the Russian Intensive Care Dataset (RICD) [21], a de-identified institutional clinical database developed and maintained by the Federal Research and Clinical Center of Intensive Care Medicine and Rehabilitology (FRCC ICMR). FRCC ICMR is a tertiary referral center specializing in the management and investigation of prolonged critical illness and chronic critical illness. RICD v3.0 contains routinely collected clinical data from patients treated at FRCC ICMR between December 2017 and September 2025. The database comprises 11 linked relational tables and includes demographic and anthropometric characteristics, patient movement within the institution, diagnoses, treatment data, laboratory results, clinical scales, continuously monitored variables, and hospitalization outcomes. Overall, the dataset contains 10,938 hospitalizations from 8135 patients, including 4527 ICU admissions among 4050 patients with at least one ICU stay.

### 2.2. Study Design and Setting

This retrospective single-center longitudinal cohort study used routinely collected real-world data from RICD v3.0. The study included adult ICU patients with chronic critical illness and evaluated the temporal patterns, burden, combinations, and pairwise concordance of CCI-related biomarker domains after CCI onset.

All patients available in the database were screened. Patients were eligible if they met the following criteria: age ≥ 18 years, first ICU admission during the corresponding hospitalization (only the first eligible hospitalization per patient was included), and at least one documented episode of inflammation, catabolism, and immunosuppression (ICS). An ICS episode was defined by the simultaneous occurrence within a 24-h period of CRP > 20 mg/L, serum albumin < 30 g/L, and an absolute lymphocyte count < 0.8 × 10^9^/L [13,22]. The 24-h window was chosen as a pragmatic and conservative criterion to ensure that the three laboratory abnormalities were truly contemporaneous, minimizing the risk of classifying patients based on abnormalities that occurred on separate days without ever coexisting. ICU readmissions were excluded. Additional exclusion criteria were an ICU length of stay < 24 h and the absence of documented ICD-10 diagnostic records.

All patients meeting the eligibility criteria were included; no formal sample size calculation was performed. The data anonymization protocol for the Russian Intensive Care Dataset was reviewed and approved by the Local Ethics Committee of the Federal Research and Clinical Center of Intensive Care Medicine and Rehabilitology (Committee Decision No. 4/23/2 dated 20 December 2023). The study was reported in accordance with the Strengthening the Reporting of Observational Studies in Epidemiology (STROBE) statement [23]; the completed checklist is provided in Supplemental Table S1.

### 2.3. Data Management

Extracted variables included demographic and anthropometric characteristics, transfer status, conditions present at ICU admission, comorbidities, neurological and organ dysfunction scores, admission laboratory parameters, requirements for mechanical ventilation and vasopressor support, lengths of stay, and ICU outcomes.

CCI onset was assigned to the first documented ICS episode. For the longitudinal analysis, the observation window extended from CCI onset to the end of the first ICU admission and was divided into daily patient-level records, with one record corresponding to one patient-day after CCI diagnosis. Daily binary statuses were generated for the CRP, lymphocyte, and albumin domains. A positive domain status was retained until a subsequent available measurement no longer met the corresponding criterion; the absence of a measurement alone did not terminate an ongoing positive interval. Domain intervals extending beyond the observation window were truncated at its boundaries, and overlapping or directly adjacent intervals were merged. A critical CCI episode began when all three domains became positive within the predefined 24-h window and continued until at least one available laboratory result no longer met its domain criterion. Overlap of all three independently propagated domain statuses without a new contemporaneous 24-h laboratory triad was not classified as a critical CCI episode. All remaining CCI patient-days were classified as belonging to non-critical CCI periods. The complete CCI course was retained for patient-level temporal summaries and swimmer plots, whereas datasets restricted to non-critical CCI periods were generated for the analyses of domain-status combinations and pairwise concordance.

Data extraction and initial database queries were performed using DB Browser for SQLite, version 3.13.1. Subsequent data restructuring and quality checks were performed in Python version: 3.11.5.

### 2.4. Statistical Analysis

Continuous variables are presented as median [interquartile range], and categorical variables as absolute numbers and percentages. No numerical laboratory values were imputed. Daily domain statuses were propagated according to the predefined episode-persistence algorithm described above.

Positive domain status was defined as CRP > 20 mg/L, absolute lymphocyte count < 0.8 × 10^9^/L, or serum albumin < 30 g/L. Domain-positive intervals were evaluated from CCI onset to the end of the first ICU admission. For each domain, the number of episodes, total positive days, durations of the first and longest episodes, and the proportion of patients with at least two episodes were calculated. Domain burden was defined as the percentage of ICU days with positive domain status after CCI onset. Patient-level trajectories were visualized using swimmer plots.

Analyses of domain-status combinations and pairwise concordance were intentionally restricted to non-critical CCI patient-days because the objective was to characterize the magnitude and structure of domain discordance outside periods of complete triad convergence. Critical CCI days were not included because all three domains are simultaneously positive during these periods by definition and therefore provide deterministic pairwise concordance. Combination frequencies were presented using bar plot.

Pairwise concordance was assessed for the CRP–lymphocyte, CRP–albumin, and lymphocyte–albumin domain pairs. Concordance was defined as simultaneous negative or positive status, and discordance as positivity of only one domain. Agreement beyond chance was quantified using Cohen’s κ with 95% confidence intervals obtained by cluster bootstrap resampling at the hospitalization level with 1000 replicates. Due to Cohen’s κ is sensitive to imbalanced marginal prevalences, the marginal prevalence of positive status was additionally reported for each domain. As a sensitivity analysis, prevalence- and bias-adjusted kappa (PABAK) was calculated as PABAK = 2P_o_ − 1, where P_o_ denotes the observed proportion of agreement. PABAK was considered a complementary agreement measure rather than a replacement for Cohen’s κ.

Associations between concurrent domain statuses were evaluated using logistic generalized estimating equation models with a binomial distribution, exchangeable working correlation structure, and clustering by hospitalization. For each domain pair, the first listed domain was modeled as the dependent variable and the second domain as the independent variable. Results are reported as odds ratios (OR) with 95% confidence intervals. Pairwise status combinations were visualized using treemaps.

All tests were two-sided, with *p* < 0.05 considered statistically significant. GEE *p* values were adjusted for three pairwise comparisons using the Holm method. Analyses were performed in Python using pandas, NumPy, statsmodels, matplotlib, and squarify.

## 3. Results

### 3.1. General Characteristics of the Cohort

**Figure 1:**
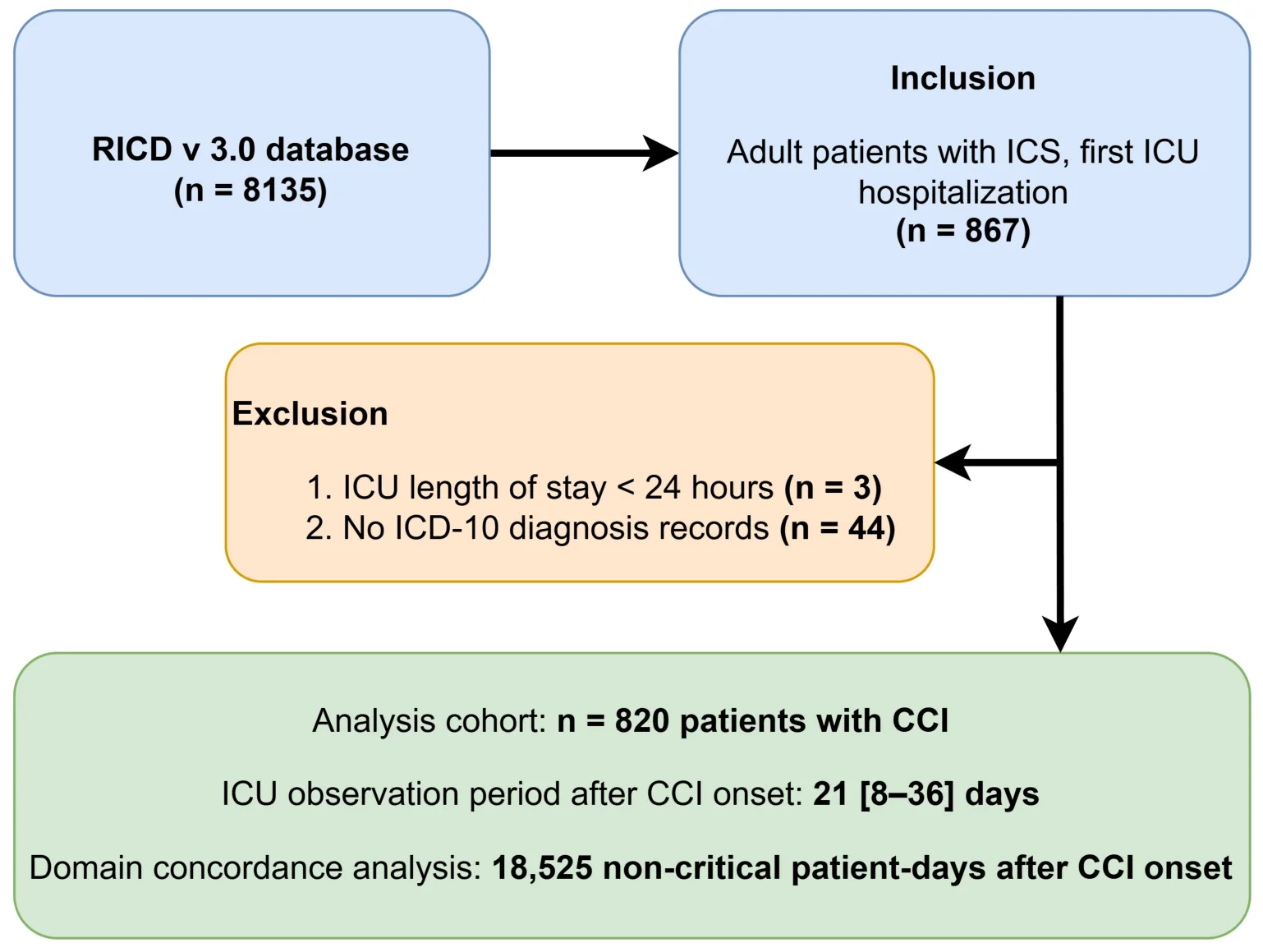
Flowchart of patient selection and inclusion in the longitudinal analysis of CCI-related biomarker domains.

Of the 8135 patients screened, 867 met the inclusion criteria. Of these, 3 were excluded because their ICU length of stay was less than 24 h, and 44 were excluded because no ICD-10 diagnostic codes were available. The final analysis included 820 patients, with a median ICU observation period after CCI onset of 21 [8–36] days. Overall, 18,525 non-critical CCI patient-days after CCI onset were included in the analyses of domain-status combinations and pairwise concordance (Figure 1).

The cohort predominantly comprised older patients with severe neurological disorders: the median age was 68 [55–77] years, and 434 patients (52.9%) were men. The most common conditions at ICU admission were ischemic stroke in 403 patients (49.1%), hemorrhagic stroke in 153 (18.7%), and traumatic brain injury in 114 (13.9%). CCI developed a median of 6 [0–17] days after ICU admission. The subsequent clinical course was characterized by prolonged ICU stays of 30 [21–55] days, a high requirement for mechanical ventilation (652 patients, 79.5%) and vasopressor support (337 patients, 41.1%), and an ICU mortality rate of 29.6% (243 patients) (Table 1).

Over 18,525 non-critical days, CRP, lymphocytes, and albumin were assessed at a median interval of 3 days (IQR 2–4 days for CRP and albumin, 1–4 days for lymphocytes). Overall cohort coverage, defined as the proportion of all patient-days with a measurement, ranged from 27.2% to 36.6%, while patient-level median coverage (the proportion of each patient’s follow-up days with assessments) varied between approximately 30% and 38%.

### 3.2. Temporal Patterns and Burden of CCI-Related Biomarker Domains

During the ICU observation period after CCI onset, the median number of positive episodes was 1 [1–1] for both the CRP and albumin domains. Positive domain status was present for 18 [7–32] days for the CRP domain and 18 [7–31] days for the albumin domain, corresponding to domain burdens of 100% [90.9–100] and 100% [91.4–100], respectively. The median durations of the first and longest positive episodes were 14 [6–26] and 16 [7–28] days for the CRP domain and 14 [5–24] and 16 [7–27] days for the albumin domain, respectively. For the lymphocyte domain, the median number of positive episodes was 1 [1–2], and 370 patients (45.1%) had two or more positive episodes, compared with 171 (20.9%) for the CRP domain and 180 (22.0%) for the albumin domain. Positive lymphocyte-domain status was present for 5 [3–9] days, corresponding to a domain burden of 34.2% [14.3–68.1]. The median durations of the first and longest positive episodes were 3 [2–4] and 4 [2–6] days, respectively (Table 2).

Patient-level trajectories in the swimmer plot showed prolonged CRP- and albumin-domain positivity and shorter, intermittent periods of lymphocyte-domain positivity (Figure 2; Supplementary Figures S1–S17).

**Figure 3:**
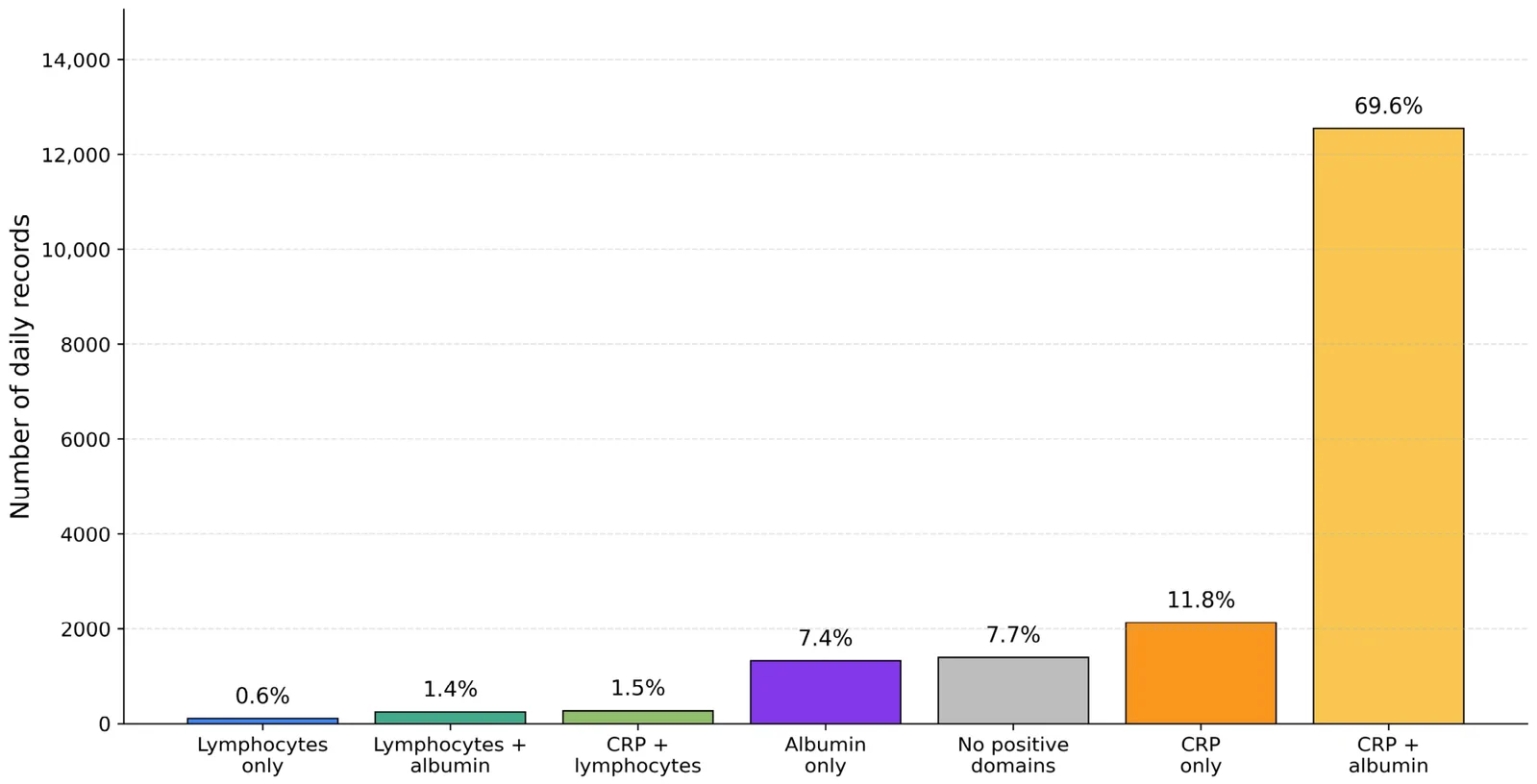
Distribution of CCI-related biomarker domain status combinations during ICU observation after CCI onset.

Among the analyzed domain-status combinations, concurrent positivity of the CRP and albumin domains was the most frequent pattern, accounting for 69.6% of observations. Isolated CRP-domain positivity accounted for 11.8%, while no positive domains were present in 7.7%. Isolated albumin-domain positivity was observed in 7.4%. Combinations involving lymphocyte-domain positivity were less frequent: concurrent CRP- and lymphocyte-domain positivity accounted for 1.5%, concurrent lymphocyte- and albumin-domain positivity for 1.4%, and isolated lymphocyte-domain positivity for 0.6% (Figure 3).

### 3.3. Pairwise Concordance Between CCI-Related Biomarker Domains

**Figure 4:**
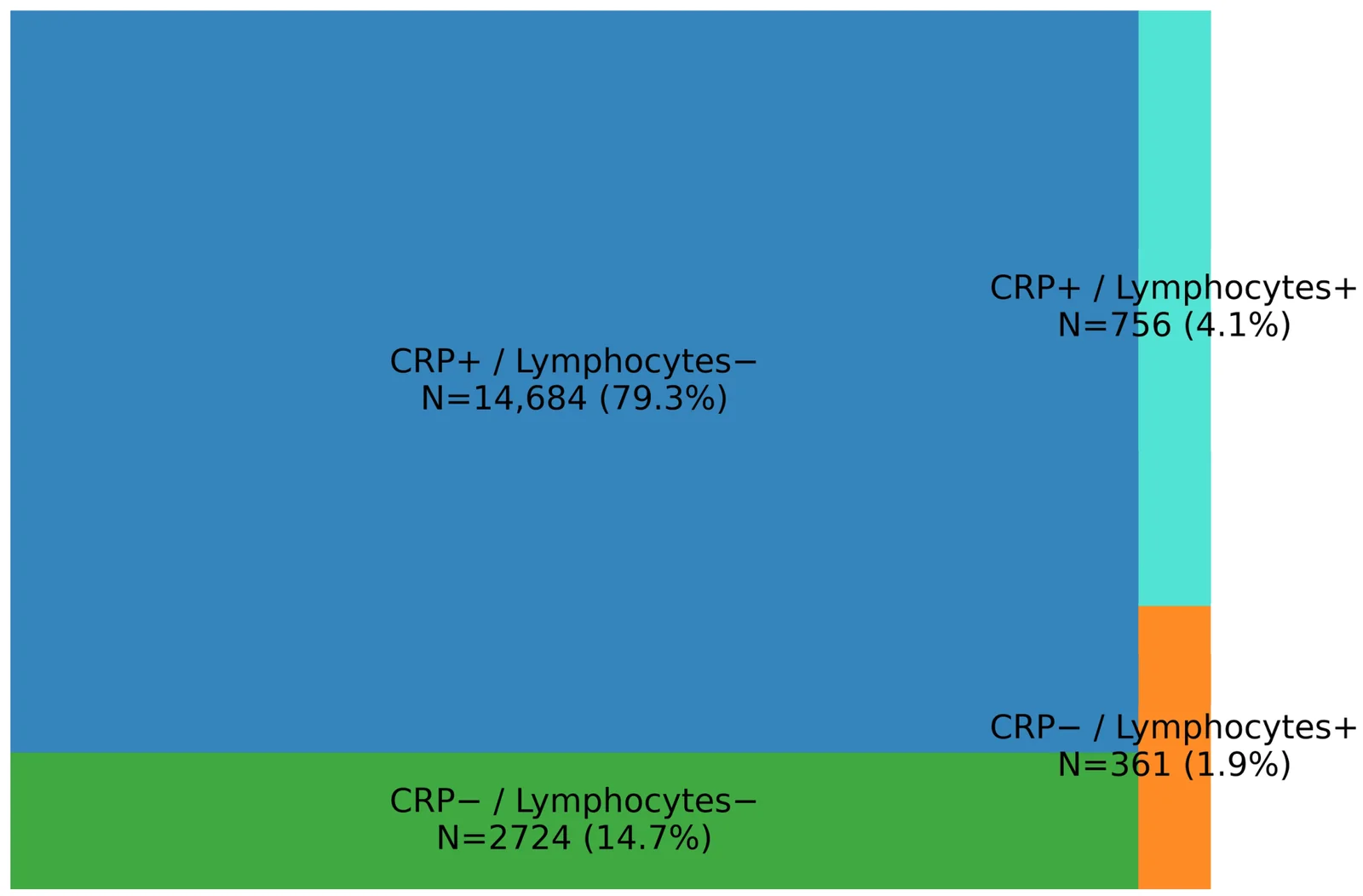
Concordance and discordance between CRP and lymphocyte domains. Notes: CRP, C-reactive protein. Positive domain status is indicated by “+”, and negative domain status by “−”.

**Figure 5:**
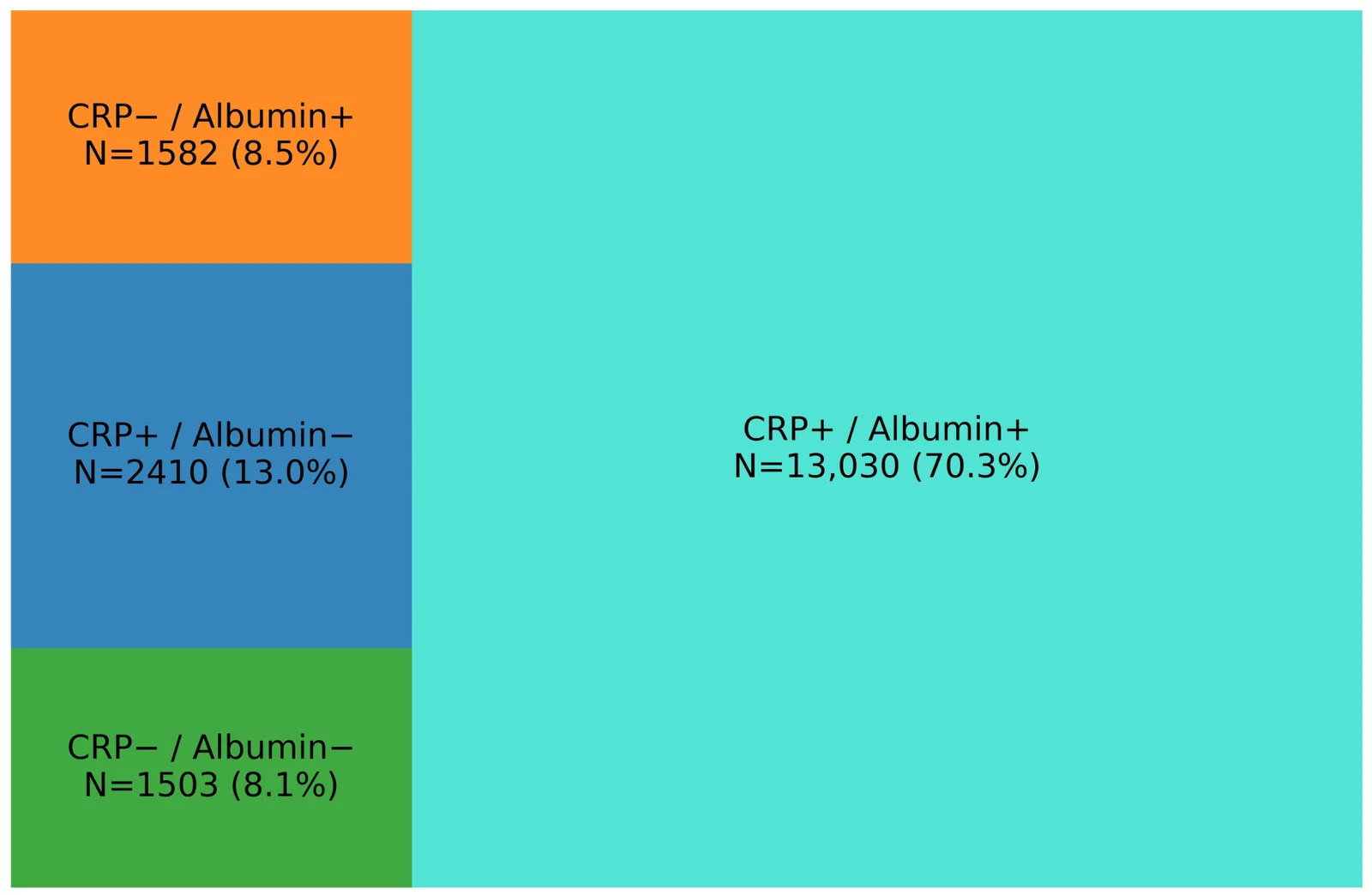
Concordance and discordance between CRP and albumin domains. Notes: CRP, C-reactive protein. Positive domain status is indicated by “+”, and negative domain status by “−”.

**Figure 6:**
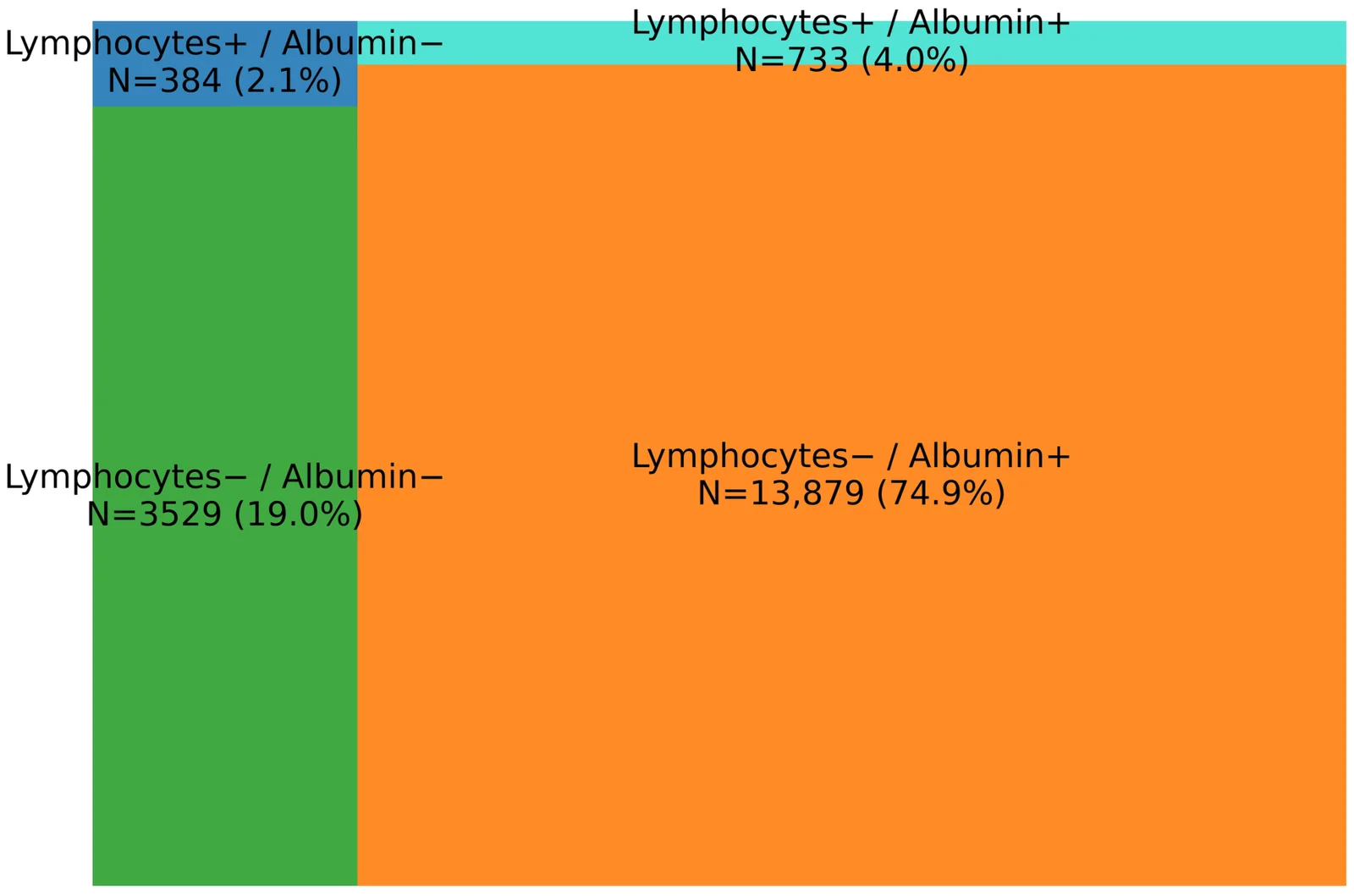
Concordance and discordance between lymphocyte and albumin domains. Notes: Positive domain status is indicated by “+”, and negative domain status by “−”.

Pairwise concordance was assessed across 18,525 non-critical CCI patient-days for each domain pair (Table 3; Figure 4, Figure 5 and Figure 6). The marginal prevalence of positive daily domain status was 83.3% (15,440/18,525) for CRP, 6.0% (1117/18,525) for lymphocytes, and 78.9% (14,612/18,525) for albumin, demonstrating marked imbalance in the marginal distributions.

The CRP and lymphocyte domains showed different daily patterns: their statuses were discordant in 15,045 patient-days (81.2%) and concordant in 3480 (18.8%). In most patient-days, the CRP domain was positive while the lymphocyte domain remained negative (14,684 patient-days, 79.3%). There was no meaningful agreement beyond chance (Cohen’s κ, −0.024; 95% CI, −0.038 to −0.012). Consistently, lymphocyte-domain positivity was associated with 40% lower odds of concurrent CRP-domain positivity in the GEE model (OR, 0.60; 95% CI, 0.43–0.86; Holm-adjusted *p* = 0.005).

The CRP and albumin domains were more frequently present together. Their daily statuses were concordant in 14,533 patient-days (78.5%) and discordant in 3992 (21.5%). The most frequent discordant combination was a positive CRP domain with a negative albumin domain, observed in 2410 patient-days (13.0%). Agreement beyond chance was low (Cohen’s κ, 0.299; 95% CI, 0.233–0.358). Albumin-domain positivity was associated with 2.71-fold higher odds of concurrent CRP-domain positivity in the GEE model (OR, 2.71; 95% CI, 2.15–3.43; Holm-adjusted *p* < 0.001).

The lymphocyte and albumin domains also showed predominantly discordant daily patterns, with discordance in 14,263 patient-days (77.0%) and concordance in 4262 (23.0%). The predominant discordant combination was a negative lymphocyte domain with a positive albumin domain, recorded in 13,879 patient-days (74.9%). There was no meaningful agreement beyond chance (Cohen’s κ, −0.021; 95% CI, −0.034 to −0.009). In the GEE model, albumin-domain positivity was associated with 51% lower odds of concurrent lymphocyte-domain positivity (OR, 0.49; 95% CI, 0.39–0.62; Holm-adjusted *p* < 0.001). In the PABAK sensitivity analysis, the estimates were −0.624 for CRP–lymphocytes, 0.569 for CRP–albumin, and −0.540 for lymphocytes–albumin. The higher PABAK estimate for the CRP–albumin pair compared with Cohen’s κ indicates the sensitivity of κ to the imbalanced marginal prevalences, whereas both lymphocyte-containing pairs remained markedly discordant under the PABAK metric.

## 4. Discussion

### 4.1. Key Findings

In a cohort of 820 patients with CCI, the longitudinal analysis of 18,525 non-critical patient-days revealed fundamentally different behavior among the three biomarker domains. The CRP and albumin domains were persistently positive throughout most of the observation period (median burden 100% for both), whereas lymphocyte-domain positivity was brief and intermittent (median burden 34.2%). Pairwise concordance showed that the CRP and lymphocyte domains were discordant in 81.2% of patient-days, with no agreement beyond chance (κ = −0.024) and a significant inverse association (OR 0.60, Holm-adjusted *p* = 0.005). A similar inverse pattern was observed between lymphocyte and albumin domains (discordance 77.0%, κ = −0.021, OR 0.49, Holm-adjusted *p* < 0.001). In marked contrast, the CRP and albumin domains were largely concordant (78.5%) with limited agreement beyond chance (κ = 0.299) and a strong positive association (OR 2.71, Holm-adjusted *p* < 0.001). The Cohen’s κ estimates should be interpreted cautiously because the marginal prevalence of daily domain positivity was markedly imbalanced, particularly for the lymphocyte domain. Cohen’s κ and PABAK are measures of agreement, whereas GEE-derived ORs quantify association between concurrent domain statuses while accounting for repeated observations within patients; consequently, their numerical estimates need not correspond directly.

### 4.2. Relationship with Previous Studies

Several lines of evidence have established that the triad of inflammation, catabolism, and immunosuppression identifies a high-risk population among critically ill patients, but the temporal relationships among its components after triad onset have remained largely unexplored.

Pei and colleagues applied group-based trajectory modelling to absolute lymphocyte counts in 10,619 critically ill patients and identified four distinct absolute lymphocyte count trajectories, demonstrating that a persistent lymphopenia endotype was associated with the highest incidence of PICS (24.9%) and hospital mortality (14.5%) [24]. While their work established that dynamic monitoring of lymphocyte counts provides superior prognostic information compared with single time-point measurements, it focused exclusively on the lymphocyte component. Our study extends this finding by simultaneously analyzing all three components of the ICS triad (CRP, lymphocytes, and albumin), revealing that the lymphocyte domain, unlike CRP and albumin, is not only intermittent but almost never occurs in isolation (0.6% of observations). This suggests that lymphopenia in CCI is predominantly superimposed on a background of pre-existing inflammation and catabolism.

Nakamura and colleagues addressed the question of CRP thresholds in PICS by applying K-means clustering to 14-day CRP trajectories in 539 prolonged-stay patients, identifying seven distinct trajectory classes and proposing a day-14 CRP cut-off of 3.0 mg/dL for PICS diagnosis [25]. While we adopted similar threshold-based definitions for domain positivity, we move beyond static or single-marker assessments by examining the simultaneous interplay of all three components throughout the ICU course. Our patient-day-level analysis (18,525 non-critical CCI days) demonstrates that patients cycle between periods of partial and complete compensation rather than remaining in a fixed state, addressing the acknowledged gap that the pathophysiological state is complex and not necessarily stable. The biomarker thresholds used in the present study are supported by the independent derivation and external validation performed by Nakamura et al. [25] Subsequent studies from our group have replicated the prognostic relevance of this biomarker-based definition of the ICS triad in separate cohorts. However, these studies were conducted within the same investigative network and therefore represent replication rather than independent external validation. Independent validation of this biomarker-based definition outside our research network remains limited and warrants further study.

Darden and colleagues took a more comprehensive approach by measuring multiple PICS-related biomarkers (inflammation, immunosuppression, stress metabolism, and angiogenesis) over 14 days in 349 surgical sepsis survivors [26]. They demonstrated that CCI patients and those with poor 1-year functional outcomes exhibited persistent elevations in IL-6, IL-8, IP-10, sPDL-1, and Ang-2, alongside lower lymphocyte counts and albumin. Our study complements their findings by demonstrating frequent concurrent positivity of the CRP and albumin domains (observed concordance 78.5%; κ = 0.299; PABAK = 0.569), whereas the lymphocyte domain showed predominantly discordant status patterns with both CRP and albumin (discordance 77.0–81.2%; PABAK −0.624 and −0.540, respectively). This pairwise concordance approach provides the first quantitative evidence that the immunosuppressive component follows a fundamentally different temporal pattern from the inflammatory and catabolic components, challenging the notion of CCI as a unified state. However, the CRP–albumin association should not necessarily be interpreted as coupling between two biologically independent processes. Given that serum albumin is a negative acute-phase reactant, hypoalbuminemia may partly reflect the same inflammatory activity captured by CRP. Thus, the observed concordance may represent a combination of inflammation–catabolism interaction and overlap in the biological information conveyed by these two biomarkers. More specific markers of catabolism are required to determine whether similar concordance exists between the underlying biological processes themselves.

Collectively, these observations provide the first evidence that the three components of the ICS triad follow temporally discordant trajectories after CCI onset, and that the lymphocyte domain represents a distinct physiological signal rather than a mere correlate of disease severity. Our findings shift the conceptualization of CCI from a static, unified state to a dynamic process in which individual domains may recover and relapse independently, with periods of complete compensation representing a potential exit from the CCI state.

### 4.3. Significance of Study Findings

The present findings have several implications that extend beyond descriptive biomarker characterization and inform both the pathophysiological model of chronic critical illness and its clinical monitoring.

First, this study provides direct evidence of temporal discordance among the three ICS components after triad onset. Although the triad is often conceptualized as a unified state, our data demonstrate that its components follow distinct temporal trajectories. The CRP and albumin domains remained persistently positive throughout most of the CCI period (median burden 100% each), whereas the lymphocyte domain was positive in only 34.2% of days, was intermittent, and recurred in 45.1% of patients. Pairwise analyses further supported this dissociation: discordance between CRP and lymphocytes occurred in 81.2% of patient-days and between lymphocytes and albumin in 77.0%, with corresponding PABAK values of −0.624 and −0.540 and inverse associations in the GEE models. Thus, CCI is not a static entity but a dynamic process in which individual domains may recover and relapse independently, creating a pattern of recurrent critical episodes when all three converge.

Second, lymphocytopenia was the least frequent domain abnormality and, notably, almost never occurred in isolation. Isolated lymphocyte domain positivity accounted for only 0.6% of observations, compared with 11.8% for isolated CRP positivity and 7.4% for isolated albumin positivity. This indicates that lymphopenia in patients with CCI predominantly arises against a background of pre-existing inflammation and catabolism, rather than as a primary event. Clinically, the emergence of lymphopenia in a patient with elevated CRP and low albumin should be regarded as a marker of ongoing triad convergence rather than an isolated laboratory finding, and warrants heightened clinical awareness.

Third, we observed episodes of complete triad compensation (periods with no positive domains) which comprised 7.7% of all observations. Whether such periods represent true resolution of CCI or transient remission remains uncertain. Our data raise the hypothesis that complete compensation may signify a potential exit from the CCI state, but this interpretation requires dedicated validation through outcome studies specifically designed to examine patient trajectories and clinical endpoints after such compensation. Should this hypothesis be confirmed, complete compensation might in future serve as a meaningful therapeutic target or a criterion for de-escalation of care, but such applications are not supported by the current data.

Taken together, these observations challenge the conventional static view of CCI and support a dynamic model characterized by intermittent and recurrent episodes of inflammation, catabolism and lymphopenia, and potential for complete, albeit poorly characterized, compensation. This framework underscores the need for component-specific longitudinal monitoring and provides a basis for future studies aimed at refining risk stratification and evaluating phase-targeted interventions.

### 4.4. Strengths and Limitations

The principal strengths of this study include its longitudinal, time-dependent analytical framework and the granularity of the data. By treating each domain as a time-varying exposure and using Cohen’s κ with cluster bootstrap resampling and GEE models, we were able to quantify pairwise concordance and domain associations with high precision while accounting for within-patient clustering. The large cohort of 820 patients with 18,525 non-critical patient-days, derived from a specialized tertiary center, provided substantial statistical power. The use of routinely available laboratory parameters (CRP, albumin, absolute lymphocyte count) enhances the clinical applicability of our findings.

Several limitations must be acknowledged. The retrospective single-center design may limit generalizability. Furthermore, our cohort was predominantly composed of patients with severe neurological injury (stroke, traumatic brain injury, and anoxic brain injury), reflecting the specific case mix of our tertiary referral center. Whether our findings apply to other CCI populations, such as those with sepsis, surgical illness, or medical critical illness, remains to be determined. Laboratory measurements were obtained according to clinical necessity rather than a fixed protocol, which could introduce variability in episode demarcation. Consequently, the irregular sampling intervals inherent to our real-world dataset preclude robust lag-based analyses such as cross-correlation or Granger causality, which would be required to establish phase-shifted asynchrony in the physiological sense. Any estimated temporal lags could reflect sampling frequency rather than true biological dynamics. We also acknowledge that defining CCI onset by the first ICS episode may introduce circularity. However, a sensitivity analysis using a clinical definition is not feasible given the lack of a universally accepted consensus definition of CCI. Additionally, we could not fully account for the potential effects of concomitant therapies (e.g., nutritional support, corticosteroid use, immunosuppressive agents, sepsis, viral infections, transfusions, renal replacement therapy, major surgery, and stress responses), which introduces a risk of residual confounding. External validation in prospective multicenter cohorts is warranted.

Importantly, our analysis relies on only three routine laboratory markers (CRP, albumin, and lymphocyte count). Although these have been validated as prognostic surrogates of the ICS triad, they cannot fully represent the complex pathophysiology of inflammation, catabolism, and immunosuppression, which involves a broad network of cytokine cascades, metabolic pathways, and immune cell interactions. Thus, our findings should be interpreted within this operational framework, and future studies using more specific biomarkers (e.g., IL-6, IL-10, sPDL-1, or lymphocyte subpopulations) are needed to confirm whether similar discordant patterns are observed at a deeper biological level.

Finally, we emphasize that this study was designed to characterize temporal patterns of biomarker domains and was not powered or intended to evaluate associations with clinical outcomes. Any statements regarding the potential clinical significance of compensation or discordance are therefore hypothesis-generating rather than evidence-based prognostic claims, and dedicated outcome-focused studies are required to establish whether these temporal patterns translate into meaningful differences in patient survival, functional recovery, or other clinically relevant endpoints.

### 4.5. Future Prospects

Our findings open several avenues for future investigation.

First, the marked temporal discordance among the triad components and the distinct temporal behaviour of the lymphocyte domain warrant prospective studies specifically designed to elucidate the determinants of lymphopenic episodes—whether they are driven by intercurrent infections, therapeutic interventions, or intrinsic immune oscillations. Furthermore, future studies with protocol-driven, standardized blood sampling schedules are essential to determine whether the observed discordance reflects true phase-shifted asynchrony, and whether changes in one domain systematically precede changes in another with a predictable time offset. Such studies would allow the application of cross-correlation and other lag-based analytical methods that are not feasible with retrospectively collected clinical data.

Second, the observation that complete triad compensation occurs in a substantial proportion of patient-days (7.7%) raises the critical question of whether such periods represent true resolution of CCI or merely transient remission. Importantly, we emphasize that our current analysis does not assess clinical outcomes, and we cannot determine whether periods of compensation are associated with improved survival, reduced relapse rates, or higher likelihood of ICU discharge. Longitudinal outcome studies specifically designed to examine patient trajectories after achieving complete compensation are therefore needed to establish the prognostic significance of this state.

Third, the inverse associations we observed between lymphocytopenia and both CRP and albumin suggest that the lymphocyte domain or the ratio of lymphocytes to inflammatory/catabolic markers may serve as a more sensitive indicator of transient clinical instability. Future research should explore the prognostic value of such composite indices and evaluate whether phase-targeted interventions (e.g., immunomodulatory therapies, nutritional optimization) can favorably modify domain dynamics and improve outcomes.

Fourth, while our analysis focused on group-level concordance patterns, substantial inter-patient heterogeneity likely exists in the degree of temporal discordance among the three domains. Future studies should quantify patient-level indices of discordance such as the proportion of days with discordant statuses, the variability of domain trajectories, or cross-correlation strength, and examine whether these measures are associated with clinical outcomes including mortality, functional recovery, and duration of organ support. Such analyses could ultimately enable the development of dynamic, individualized risk stratification tools that reflect not only the presence of the triad but also its temporal coherence.

## 5. Conclusions

This study demonstrates that after CCI onset, the three components of the ICS triad follow distinct, temporally discordant trajectories, challenging the conventional static view of CCI as a unified state. Periods of complete compensation of all three domains were observed, raising the hypothesis that such compensation may represent a potential exit from the CCI state. However, we emphasize that this hypothesis requires dedicated outcome-based studies to determine the clinical significance of these observations.

## Figures and Tables

**Figure 2:**
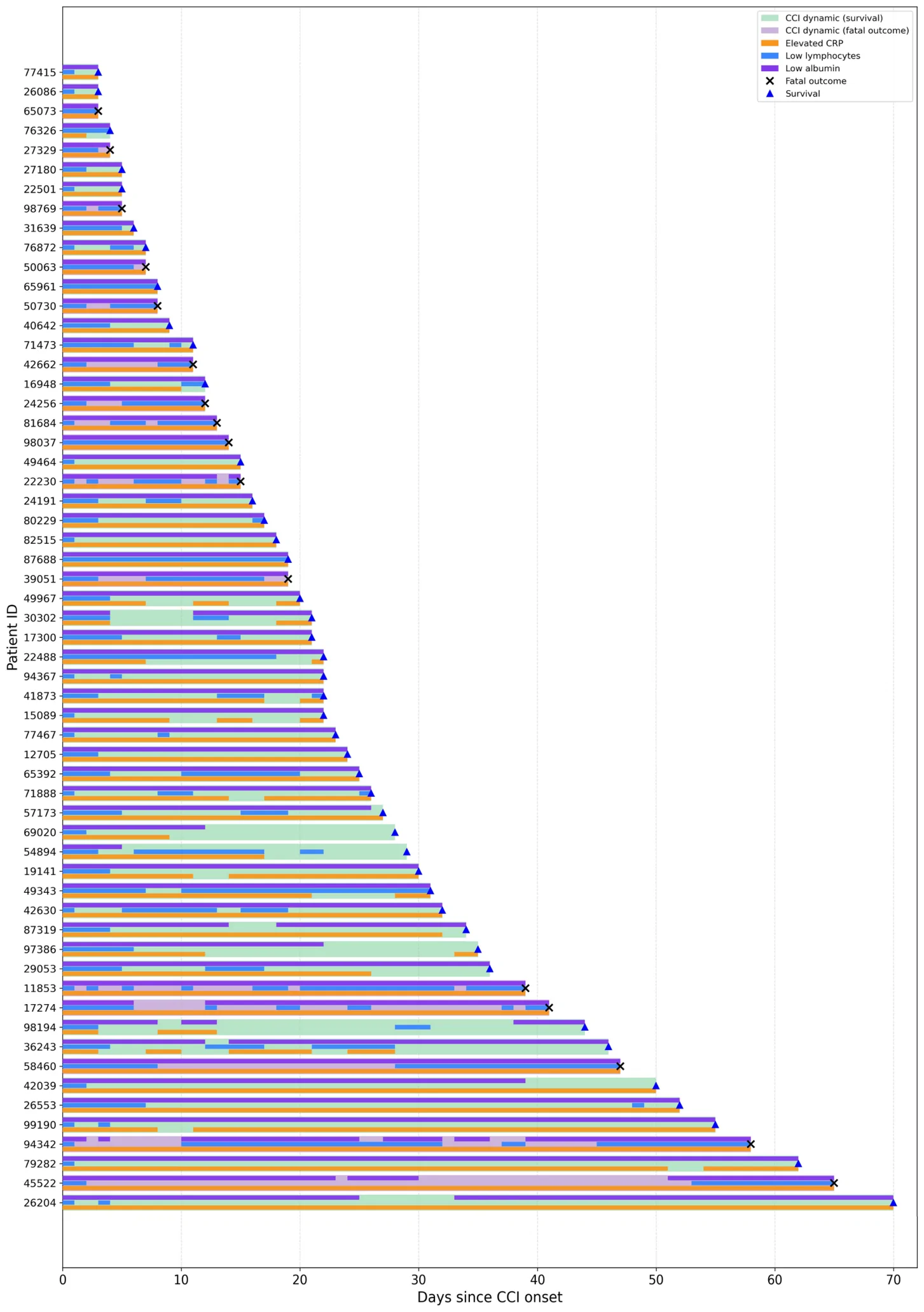
Swimmer plot of CCI-related biomarker domain trajectories after CCI onset.

**Table 1:** Baseline characteristics, admission laboratory profile, and clinical course of the study cohort.

Parameter	Patients with CCI (N = 820)
Demographic and admission characteristics	
Male sex, *n* (%)	434 (52.9%)
Age, years	68 [55–77]
BMI, kg/m^2^	25.1 [22.1–29.3]
Transferred from another hospital, *n* (%)	773 (94.3%)
Conditions at ICU admission	
Ischemic stroke, *n* (%)	403 (49.1%)
Hemorrhagic stroke, *n* (%)	153 (18.7%)
Traumatic brain injury, *n* (%)	114 (13.9%)
Anoxic brain injury, *n* (%)	59 (7.2%)
Pneumonia, *n* (%)	474 (57.8%)
Comorbidities	
Arterial hypertension, *n* (%)	597 (72.8%)
Coronary artery disease, *n* (%)	340 (41.5%)
CHF, *n* (%)	190 (23.2%)
Type 2 diabetes, *n* (%)	88 (10.7%)
CKD, *n* (%)	77 (9.4%)
Neurological and organ dysfunction scales at ICU admission	
GCS, points	10 [8–13]
FOUR, points	13 [10–15]
SOFA-2, points	3 [2–5]
Laboratory parameters at ICU admission	
Hemoglobin, g/L	106 [94–121]
WBC, ×10^9^/L	9.6 [7.2–12.8]
Lymphocytes, ×10^9^/L	0.90 [0.60–1.30]
Platelets, ×10^9^/L	251 [188–343]
Albumin, g/L	28.2 [24.9–31.8]
CRP, mg/L	69.5 [36.8–132.8]
Creatinine, µmol/L	77.8 [58.9–111.5]
Urea, mmol/L	7.4 [4.7–12.6]
CCI characteristics and outcomes	
Time from ICU admission to CCI onset, days	6 [0–17]
ICU observation period after CCI onset, days	21 [8–36]
Number of critical CCI episodes	1 [1–2]
Total duration of critical CCI episodes, days	4 [2–8]
Need for MV, *n* (%)	652 (79.5%)
Need for vasopressor support, *n* (%)	337 (41.1%)
ICU mortality, *n* (%)	243 (29.6%)
ICU length of stay, days	30 [21–55]
Hospital length of stay, days	39 [22–67]

Notes: BMI, body mass index; CCI, chronic critical illness; CHF, chronic heart failure; CKD, chronic kidney disease; CRP, C-reactive protein; FOUR, Full Outline of UnResponsiveness; GCS, Glasgow Coma Scale; ICU, intensive care unit; MV, mechanical ventilation; SOFA-2, Sequential Organ Failure Assessment 2; WBC, white blood cell count.

**Table 2:** Temporal characteristics and burden of CCI-related biomarker domains.

Parameter	CRP Domain	Lymphocyte Domain	Albumin Domain
Number of positive episodes per patient	1 [1–1]	1 [1–2]	1 [1–1]
Patients with ≥2 positive episodes, *n* (%)	171 (20.9%)	370 (45.1%)	180 (22.0%)
Days with positive domain status, days	18 [7–32]	5 [3–9]	18 [7–31]
Domain burden, %	100 [90.9–100]	34.2 [14.3–68.1]	100 [91.4–100]
Duration of first positive episode, days	14 [6–26]	3 [2–4]	14 [5–24]
Duration of longest positive episode, days	16 [7–28]	4 [2–6]	16 [7–27]

Notes: Domain burden was calculated as the percentage of ICU days with positive domain status after CCI onset; CCI, chronic critical illness; CRP, C-reactive protein; ICU, intensive care unit.

**Table 3:** Pairwise concordance and discordance between CCI-related biomarker domains.

Domain Pair	CRP Domain vs. Lymphocyte Domain	CRP Domain vs. Albumin Domain	Lymphocyte Domain vs. Albumin Domain
Concordant records, *n* (%)	3480 (18.8%)	14,533 (78.5%)	4262 (23.0%)
Discordant records, *n* (%)	15,045 (81.2%)	3992 (21.5%)	14,263 (77.0%)
Predominant discordant pattern, n (% of all records)	CRP+/lymphocytes−: 14,684 (79.3%)	CRP+/albumin−: 2410 (13.0%)	lymphocytes−/albumin+: 13,879 (74.9%)
Cohen’s κ (95% CI)	−0.024 [−0.038; −0.012]	0.299 [0.233; 0.358]	−0.021 [−0.034; −0.009]
PABAK	−0.624	0.569	−0.540
GEE OR (95% CI)	0.60 [0.43; 0.86]	2.71 [2.15; 3.43]	0.49 [0.39; 0.62]
GEE *p* value, Holm-adjusted	0.005	<0.001	<0.001

Notes: Data are non-critical CCI patient-days with available paired domain statuses (*n* = 18,525 for each domain pair); CCI, chronic critical illness; CI, confidence interval; CRP, C-reactive protein; GEE, generalized estimating equations; OR, odds ratio; PABAK, prevalence- and bias-adjusted kappa. GEE *p* values were adjusted for three pairwise comparisons using the Holm method.

## Data Availability

Publicly and partially available datasets were analyzed in this study. The RICD can be obtained upon request at https://fnkcrr-database.ru/ (accessed on 1 July 2026).

## Supplementary Materials

The following supporting information can be downloaded at: https://www.mdpi.com/article/10.3390/medicina62091686/s1, Supplemental Table S1: STROBE Statement—Checklist; Supplementary Figure S1: Swimmer plot of CCI-related biomarker domain trajectories after CCI onset (part 1); Supplementary Figure S2: Swimmer plot of CCI-related biomarker domain trajectories after CCI onset (part 2); Supplementary Figure S3: Swimmer plot of CCI-related biomarker domain trajectories after CCI onset (part 3); Supplementary Figure S4: Swimmer plot of CCI-related biomarker domain trajectories after CCI onset (part 4); Supplementary Figure S5: Swimmer plot of CCI-related biomarker domain trajectories after CCI onset (part 5); Supplementary Figure S6: Swimmer plot of CCI-related biomarker domain trajectories after CCI onset (part 6); Supplementary Figure S7: Swimmer plot of CCI-related biomarker domain trajectories after CCI onset (part 7); Supplementary Figure S8: Swimmer plot of CCI-related biomarker domain trajectories after CCI onset (part 8); Supplementary Figure S9: Swimmer plot of CCI-related biomarker domain trajectories after CCI onset (part 9); Supplementary Figure S10: Swimmer plot of CCI-related biomarker domain trajectories after CCI onset (part 10); Supplementary Figure S11: Swimmer plot of CCI-related biomarker domain trajectories after CCI onset (part 11); Supplementary Figure S12: Swimmer plot of CCI-related biomarker domain trajectories after CCI onset (part 12); Supplementary Figure S13: Swimmer plot of CCI-related biomarker domain trajectories after CCI onset (part 13); Supplementary Figure S14: Swimmer plot of CCI-related biomarker domain trajectories after CCI onset (part 14); Supplementary Figure S15: Swimmer plot of CCI-related biomarker domain trajectories after CCI onset (part 15); Supplementary Figure S16: Swimmer plot of CCI-related biomarker domain trajectories after CCI onset (part 16); Supplementary Figure S17: Swimmer plot of CCI-related biomarker domain trajectories after CCI onset (part 17).

## Abbreviations

The following abbreviations are used in this manuscript:

BMI	Body mass index
CCI	Chronic critical illness
CHF	Chronic heart failure
CI	Confidence interval
CKD	Chronic kidney disease
CRP	C-reactive protein
FOUR	Full Outline of UnResponsiveness
FRCC ICMR	Federal Research and Clinical Center of Intensive Care Medicine and Rehabilitology
GCS	Glasgow Coma Scale
GEE	Generalized estimating equations
ICD-10	International Classification of Diseases, 10th Revision
ICU	Intensive care unit
ICS	Inflammation, catabolism, and immunosuppression
IQR	Interquartile range
MV	Mechanical ventilation
OR	Odds ratio
RICD	Russian Intensive Care Dataset
SOFA-2	Sequential Organ Failure Assessment 2
STROBE	Strengthening the Reporting of Observational Studies in Epidemiology
WBC	White blood cell count
