# Supplementary material for: Temporal Discordance of the Inflammation, Catabolism and Immunosuppression Triad in Chronic Critical Illness
Source: Medicina (Kaunas). 2026 Sep 3;62(9):1686. doi: 10.3390/medicina62091686 (PMC13609605; doi:10.3390/medicina62091686)

Supplementary Information

Temporal Discordance of the Inflammation, Catabolism and Immunosuppression Triad in Chronic Critical Illness

Valery Likhvantsev [1,2,*], Levan Berikashvili [1], Petr Polyakov [1], Mikhail Yadgarov [1], Artem Kuzovlev [1], Alexey Yakovlev [1] and Andrey Grechko [1]

[1] Department of Clinical Trials, Federal Research and Clinical Center of Intensive Care Medicine and Rehabilitology, 107031 Moscow, Russia; lberikashvili@fnkcrr.ru (L.B.); p.polyakov@fnkcrr.ru (P.P.); myadgarov@fnkcrr.ru (M.Y.); artem_kuzovlev@fnkcrr.ru (A.K.); ayakovlev@fnkcrr.ru (A.Y.); fnkcrr@fnkcrr.ru (A.G.)

[2] Department of Anesthesiology and Intensive Care, I.M. Sechenov First Moscow State Medical University of the Russian Ministry of Health (Sechenov University), 119048 Moscow, Russia

* Correspondence: lik0704@gmail.com

Contents

Supplemental Table S1. STROBE Statement—Checklist. 3

Supplementary Figure S1. Swimmer plot of CCI-related biomarker domain trajectories after CCI onset (part 1). 6

Supplementary Figure S2. Swimmer plot of CCI-related biomarker domain trajectories after CCI onset (part 2). 7

Supplementary Figure S3. Swimmer plot of CCI-related biomarker domain trajectories after CCI onset (part 3). 8

Supplementary Figure S4. Swimmer plot of CCI-related biomarker domain trajectories after CCI onset (part 4). 9

Supplementary Figure S5. Swimmer plot of CCI-related biomarker domain trajectories after CCI onset (part 5). 10

Supplementary Figure S6. Swimmer plot of CCI-related biomarker domain trajectories after CCI onset (part 6). 11

Supplementary Figure S7. Swimmer plot of CCI-related biomarker domain trajectories after CCI onset (part 7). 12

Supplementary Figure S8. Swimmer plot of CCI-related biomarker domain trajectories after CCI onset (part 8). 13

Supplementary Figure S9. Swimmer plot of CCI-related biomarker domain trajectories after CCI onset (part 9). 14

Supplementary Figure S10. Swimmer plot of CCI-related biomarker domain trajectories after CCI onset (part 10)....... 15

Supplementary Figure S11. Swimmer plot of CCI-related biomarker domain trajectories after CCI onset (part 11)....... 16

Supplementary Figure S12. Swimmer plot of CCI-related biomarker domain trajectories after CCI onset (part 12)....... 17

Supplementary Figure S13. Swimmer plot of CCI-related biomarker domain trajectories after CCI onset (part 13)....... 18

Supplementary Figure S14. Swimmer plot of CCI-related biomarker domain trajectories after CCI onset (part 14)....... 19

Supplementary Figure S15. Swimmer plot of CCI-related biomarker domain trajectories after CCI onset (part 15)....... 20

Supplementary Figure S16. Swimmer plot of CCI-related biomarker domain trajectories after CCI onset (part 16)....... 21

Supplementary Figure S17. Swimmer plot of CCI-related biomarker domain trajectories after CCI onset (part 17)....... 22

Supplemental Table S1. STROBE Statement—Checklist.

	Item No	Recommendation	Page No. (Section(s))
**Title and abstract**	1	(*a*) Indicate the study's design with a commonly used term in the title or the abstract	Abstract — Materials and Methods
		(*b*) Provide in the abstract an informative and balanced summary of what was done and what was found	Abstract
**Introduction**			
Background/rationale	2	Explain the scientific background and rationale for the investigation being reported	1. Introduction
Objectives	3	State specific objectives, including any prespecified hypotheses	1. Introduction
**Methods**			
Study design	4	Present key elements of study design early in the paper	2.2. Study design and setting
Setting	5	Describe the setting, locations, and relevant dates, including periods of recruitment, exposure, follow-up, and data collection	2.1. Source of data; 2.2. Study design and setting
Participants	6	(*a*) Give the eligibility criteria, and the sources and methods of selection of participants. Describe methods of follow-up	2.1. Source of data; 2.2. Study design and setting; 2.3. Data management
		(*b*) For matched studies, give matching criteria and number of exposed and unexposed	Not applicable — this was not a matched study
Variables	7	Clearly define all outcomes, exposures, predictors, potential confounders, and effect modifiers. Give diagnostic criteria, if applicable	2.2. Study design and setting; 2.3. Data management; 2.4. Statistical analysis
Data sources/ measurement	8*	For each variable of interest, give sources of data and details of methods of assessment (measurement). Describe comparability of assessment methods if there is more than one group	2.1. Source of data; 2.2. Study design and setting; 2.3. Data management; 2.4. Statistical analysis; 4.4. Strengths and limitations
Bias	9	Describe any efforts to address potential sources of bias	2.2. Study design and setting; 2.3. Data management; 2.4. Statistical analysis; 4.4. Strengths and limitations
Study size	10	Explain how the study size was arrived at	2.2. Study design and setting

Quantitative variables	11	Explain how quantitative variables were handled in the analyses. If applicable, describe which groupings were chosen and why	2.3. Data management; 2.4. Statistical analysis
Statistical methods	12	(*a*) Describe all statistical methods, including those used to control for confounding	2.4. Statistical analysis
		(*b*) Describe any methods used to examine subgroups and interactions	Not applicable — no subgroup or interaction analyses were performed
		(*c*) Explain how missing data were addressed	2.3. Data management; 2.4. Statistical analysis
		(*d*) If applicable, explain how loss to follow-up was addressed	Not applicable — follow-up was defined from CCI onset to the end of the first ICU admission using routinely collected data; 2.3. Data management
		(*e*) Describe any sensitivity analyses	Not reported — no sensitivity analyses were performed
**Results**			
Participants	13*	(a) Report numbers of individuals at each stage of study—e.g., numbers potentially eligible, examined for eligibility, confirmed eligible, included in the study, completing follow-up, and analysed	3.1. General characteristics of the cohort; Figure 1
		(b) Give reasons for non-participation at each stage	3.1. General characteristics of the cohort; Figure 1
		(c) Consider use of a flow diagram	3.1. General characteristics of the cohort; Figure 1
Descriptive data	14*	(a) Give characteristics of study participants (e.g., demographic, clinical, social) and information on exposures and potential confounders	3.1. General characteristics of the cohort; Table 1
		(b) Indicate number of participants with missing data for each variable of interest	Not reported — variable-specific numbers of participants with missing data are not provided
		(c) Summarise follow-up time (e.g., average and total amount)	3.1. General characteristics of the cohort; Table 1
Outcome data	15*	Report numbers of outcome events or summary measures over time	3.1. General characteristics of the cohort; 3.2. Temporal patterns and burden of CCI-related biomarker domains; 3.3. Pairwise concordance between CCI-related biomarker domains; Tables 1–3; Figures 2–6

Main results	16	(*a*) Give unadjusted estimates and, if applicable, confounder-adjusted estimates and their precision (e.g., 95% confidence interval). Make clear which confounders were adjusted for and why they were included	2.4. Statistical analysis; 3.3. Pairwise concordance between CCI-related biomarker domains; Table 3
		(*b*) Report category boundaries when continuous variables were categorised	2.2. Study design and setting; 2.4. Statistical analysis
		(*c*) If relevant, consider translating estimates of relative risk into absolute risk for a meaningful time period	Not applicable — relative-risk estimates were not calculated
Other analyses	17	Report other analyses done—e.g., analyses of subgroups and interactions, and sensitivity analyses	Not applicable — no subgroup, interaction, or sensitivity analyses were performed
**Discussion**			
Key results	18	Summarise key results with reference to study objectives	4.1. Key findings; 5. Conclusions
Limitations	19	Discuss limitations of the study, taking into account sources of potential bias or imprecision. Discuss both direction and magnitude of any potential bias	4.4. Strengths and limitations
Interpretation	20	Give a cautious overall interpretation of results considering objectives, limitations, multiplicity of analyses, results from similar studies, and other relevant evidence	4.2. Relationship with previous studies; 4.3. Significance of study findings; 4.4. Strengths and limitations; 5. Conclusions
Generalisability	21	Discuss the generalisability (external validity) of the study results	4.4. Strengths and limitations
**Other information**			
Funding	22	Give the source of funding and the role of the funders for the present study and, if applicable, for the original study on which the present article is based	Funding

*Give information separately for exposed and unexposed groups.

Note: An Explanation and Elaboration article discusses each checklist item and gives methodological background and published examples of transparent reporting. The STROBE checklist is best used in conjunction with this article (freely available on the Web sites of PLoS Medicine at http://www.plosmedicine.org/, Annals of Internal Medicine at http://www.annals.org/, and Epidemiology at http://www.epidem.com/). Information on the STROBE Initiative is available at http://www.strobe-statement.org.

Supplementary Figure S1. Swimmer plot of CCI-related biomarker domain trajectories after CCI onset (part 1).

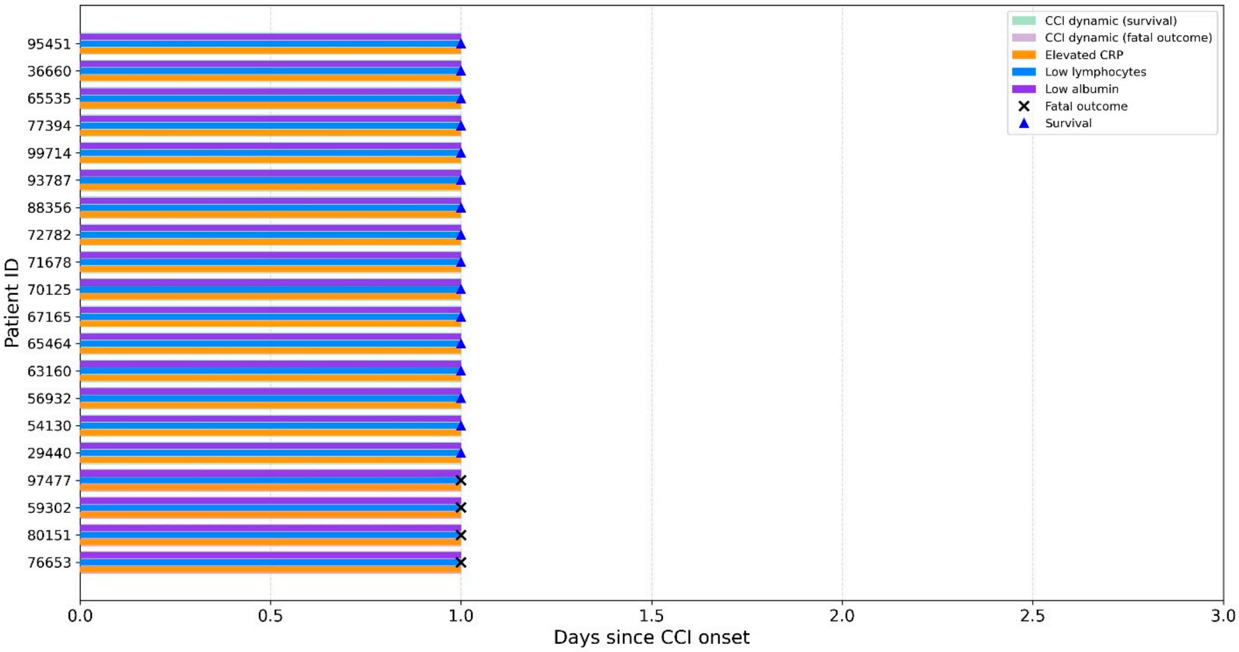

Supplementary Figure S2. Swimmer plot of CCI-related biomarker domain trajectories after CCI onset (part 2).

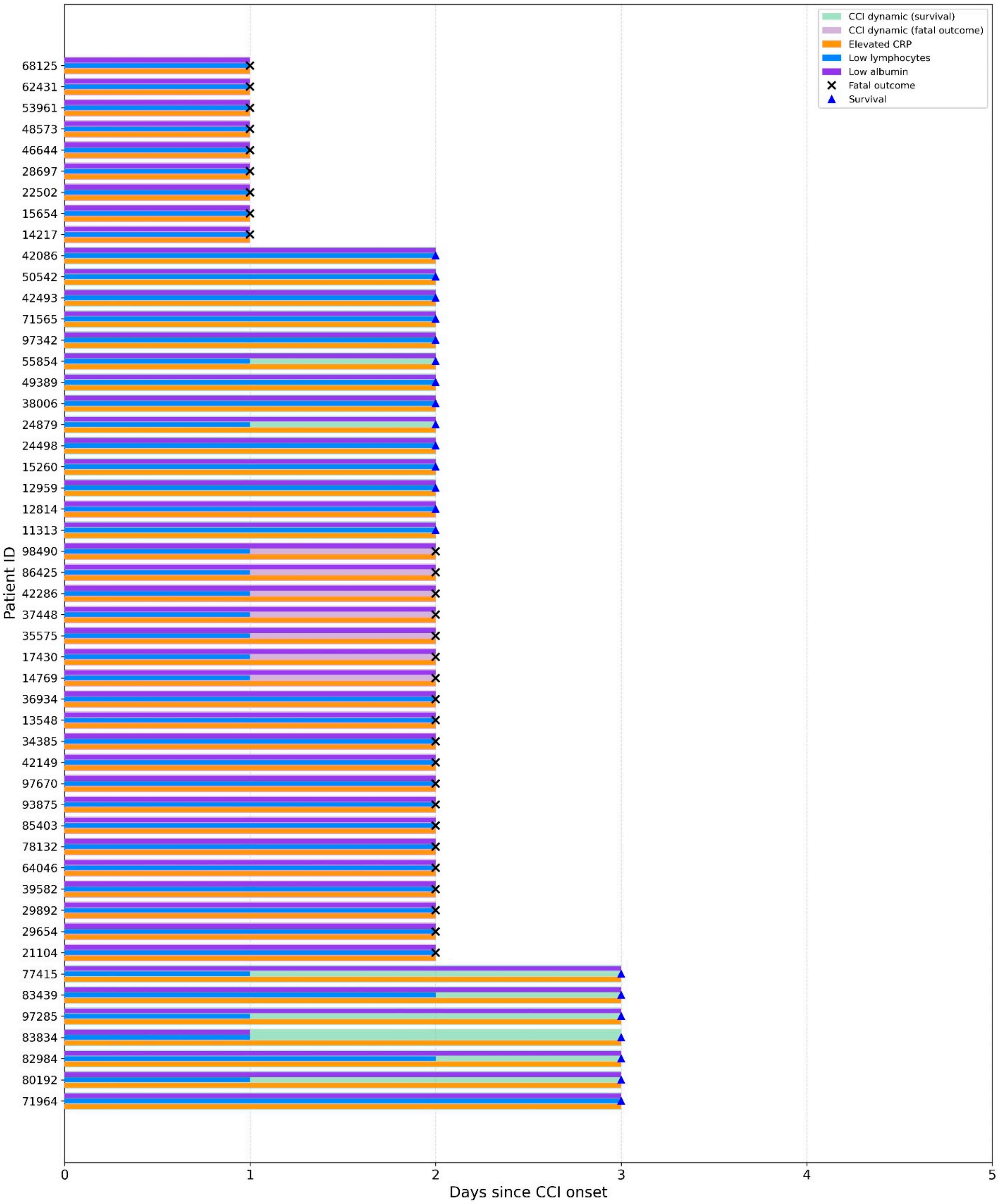

Supplementary Figure S3. Swimmer plot of CCI-related biomarker domain trajectories after CCI onset (part 3).

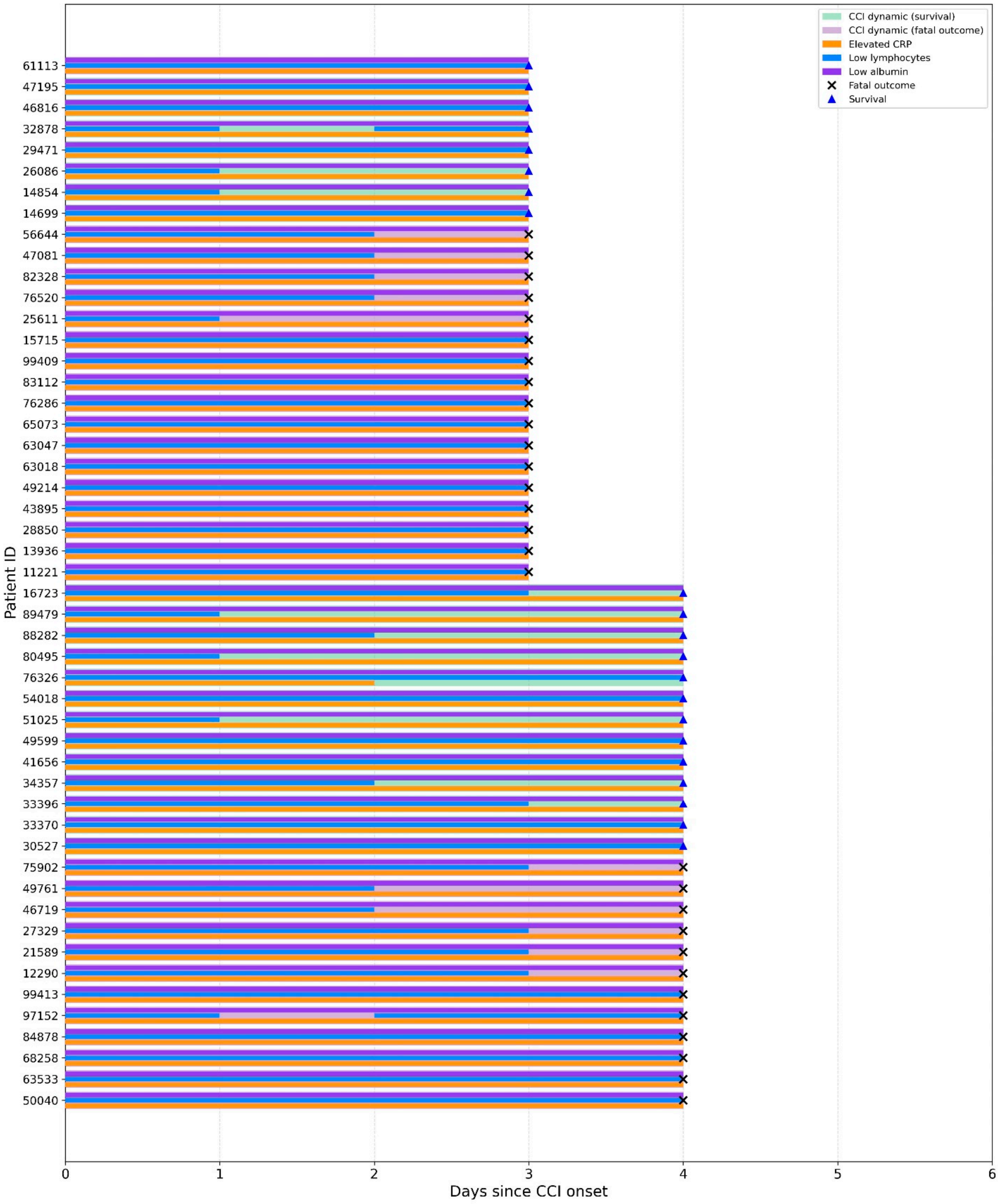

Supplementary Figure S4. Swimmer plot of CCI-related biomarker domain trajectories after CCI onset (part 4).

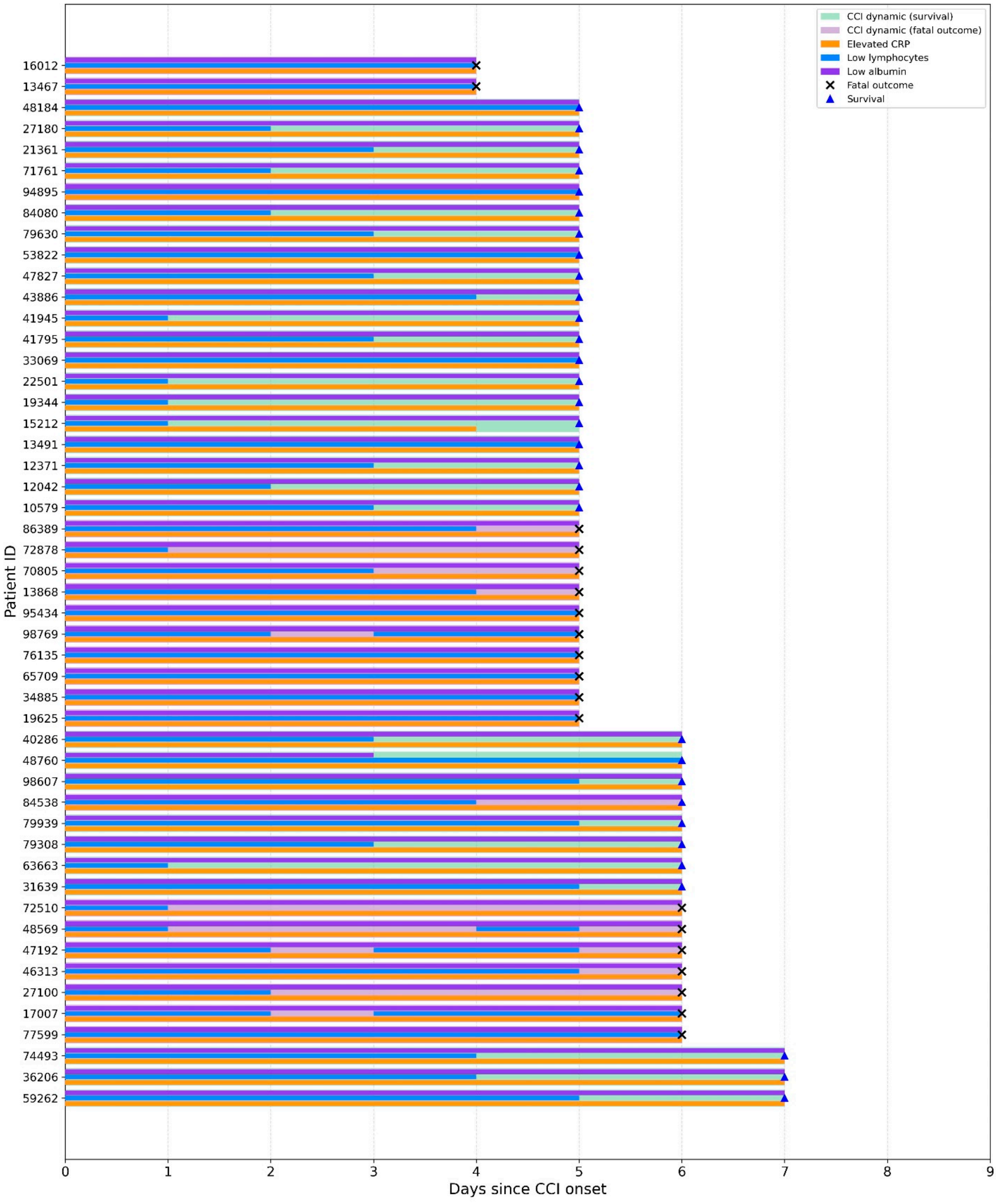

Supplementary Figure S5. Swimmer plot of CCI-related biomarker domain trajectories after CCI onset (part 5).

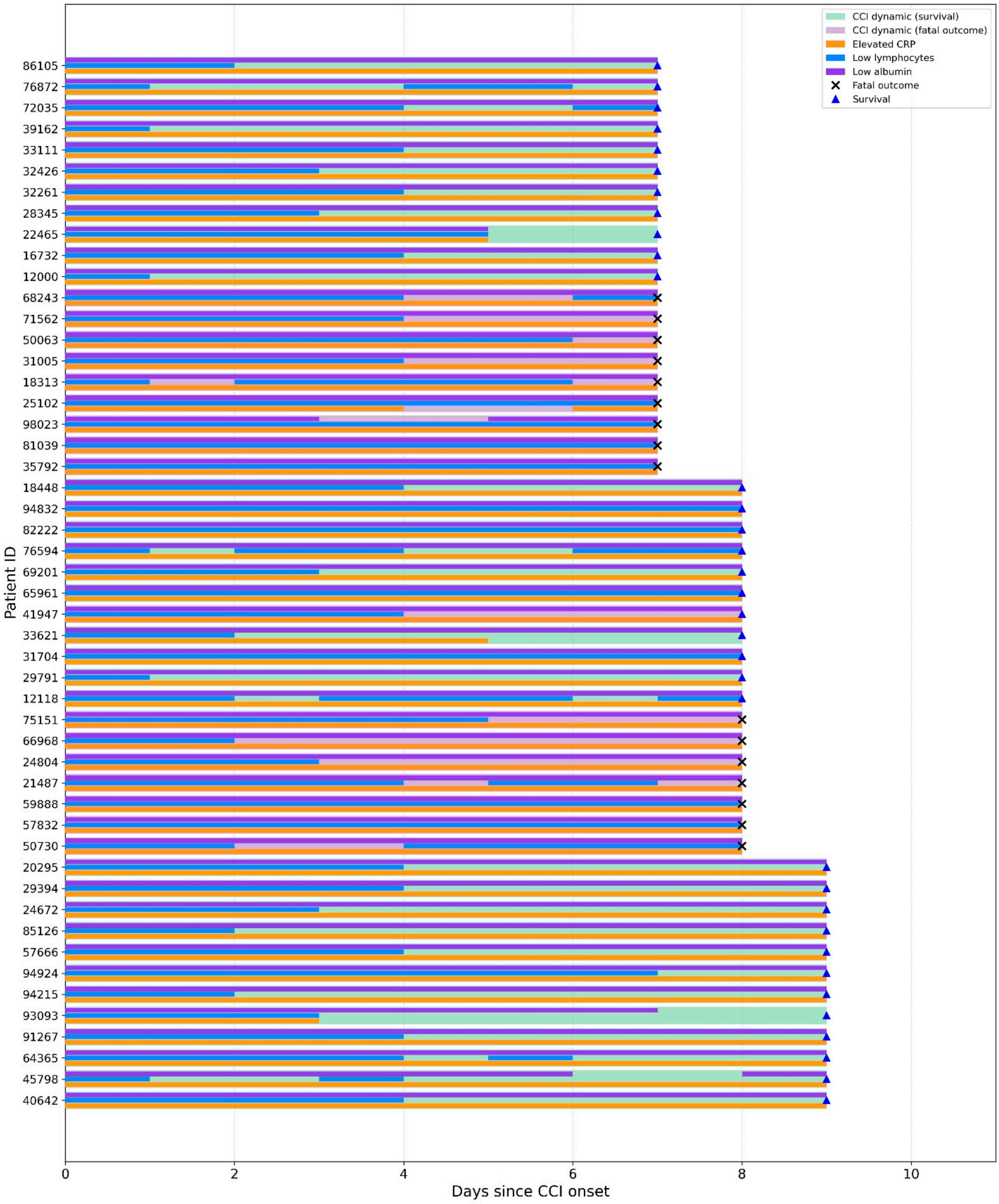

Supplementary Figure S6. Swimmer plot of CCI-related biomarker domain trajectories after CCI onset (part 6).

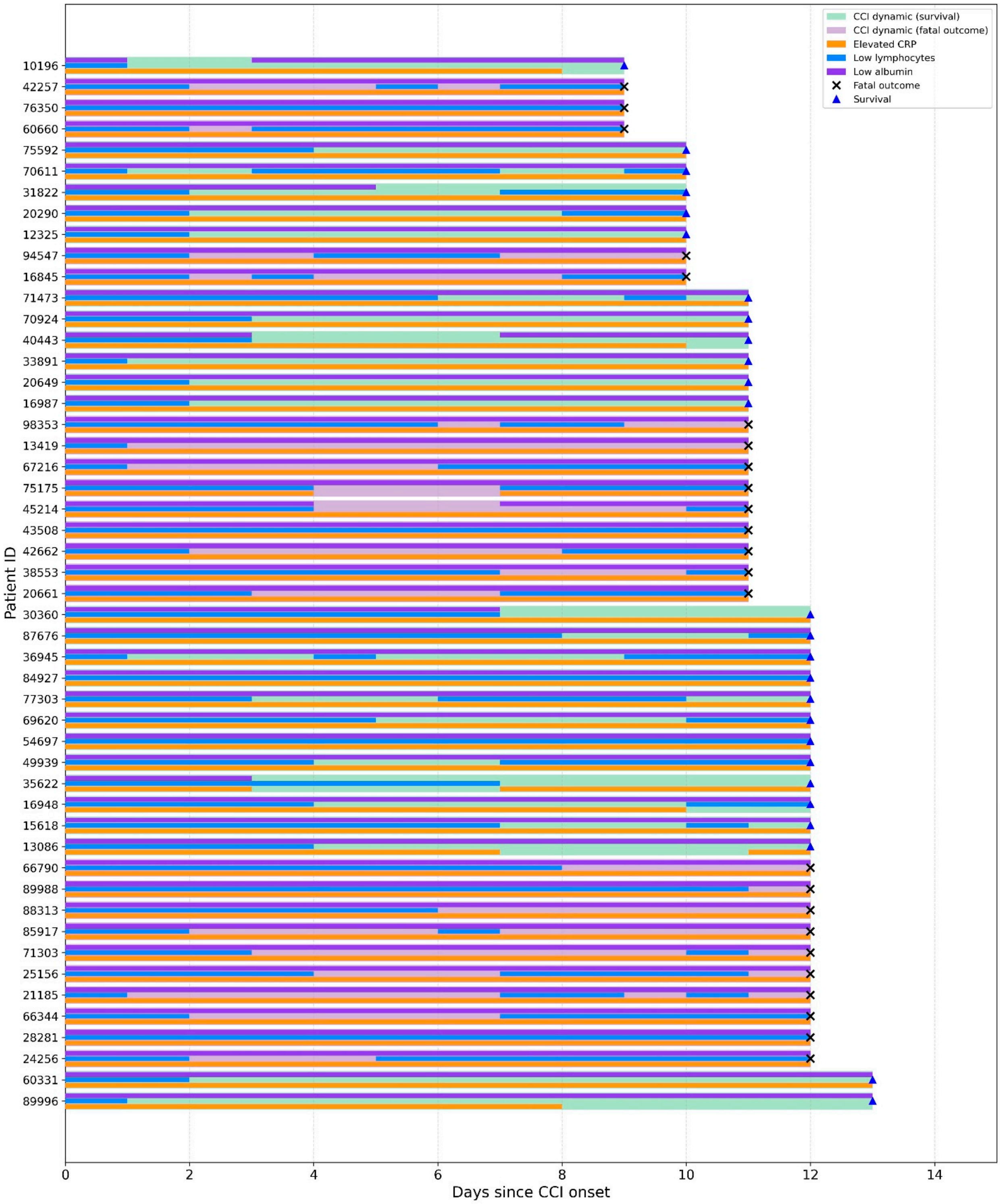

Supplementary Figure S7. Swimmer plot of CCI-related biomarker domain trajectories after CCI onset (part 7).

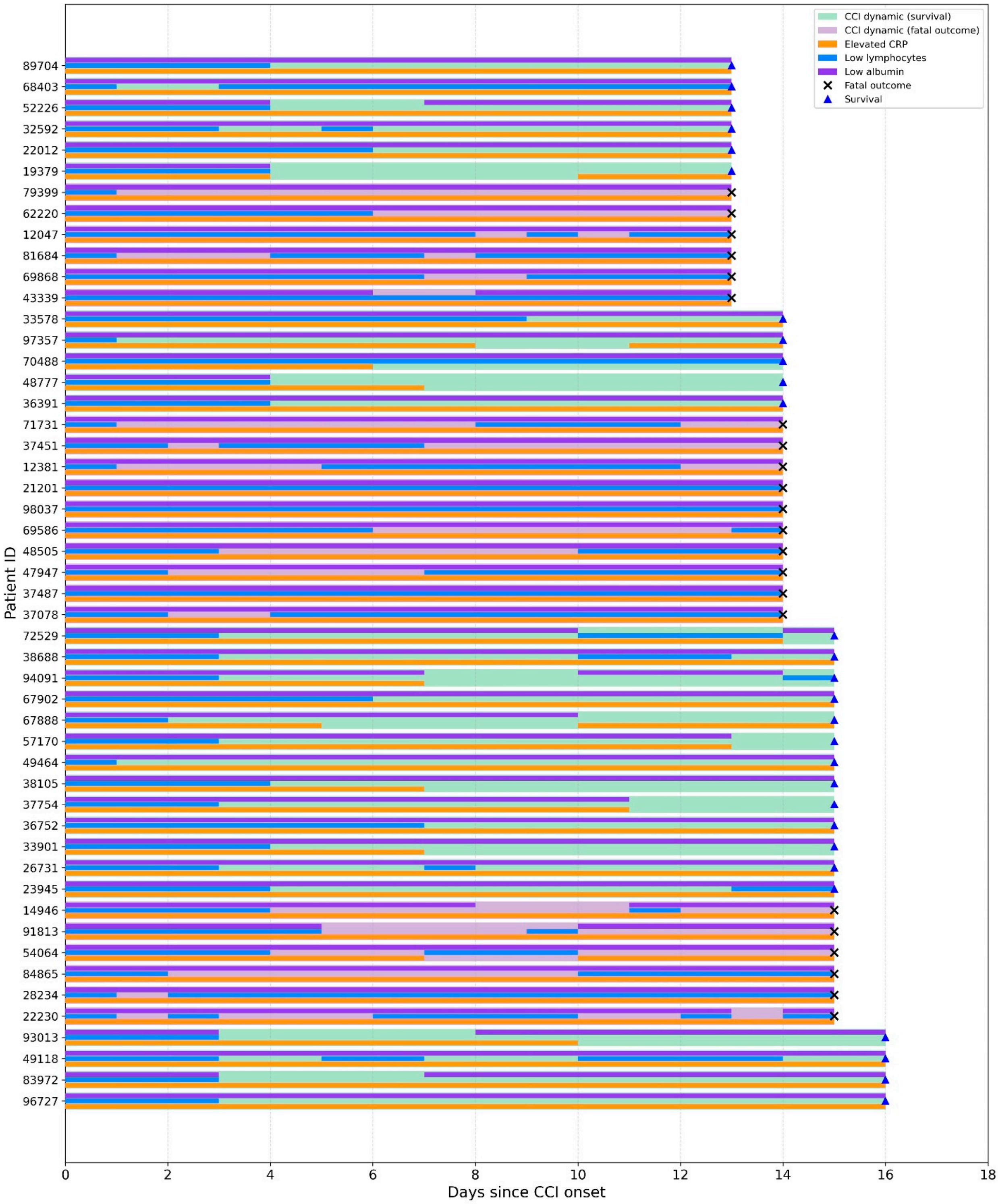

Supplementary Figure S8. Swimmer plot of CCI-related biomarker domain trajectories after CCI onset (part 8).

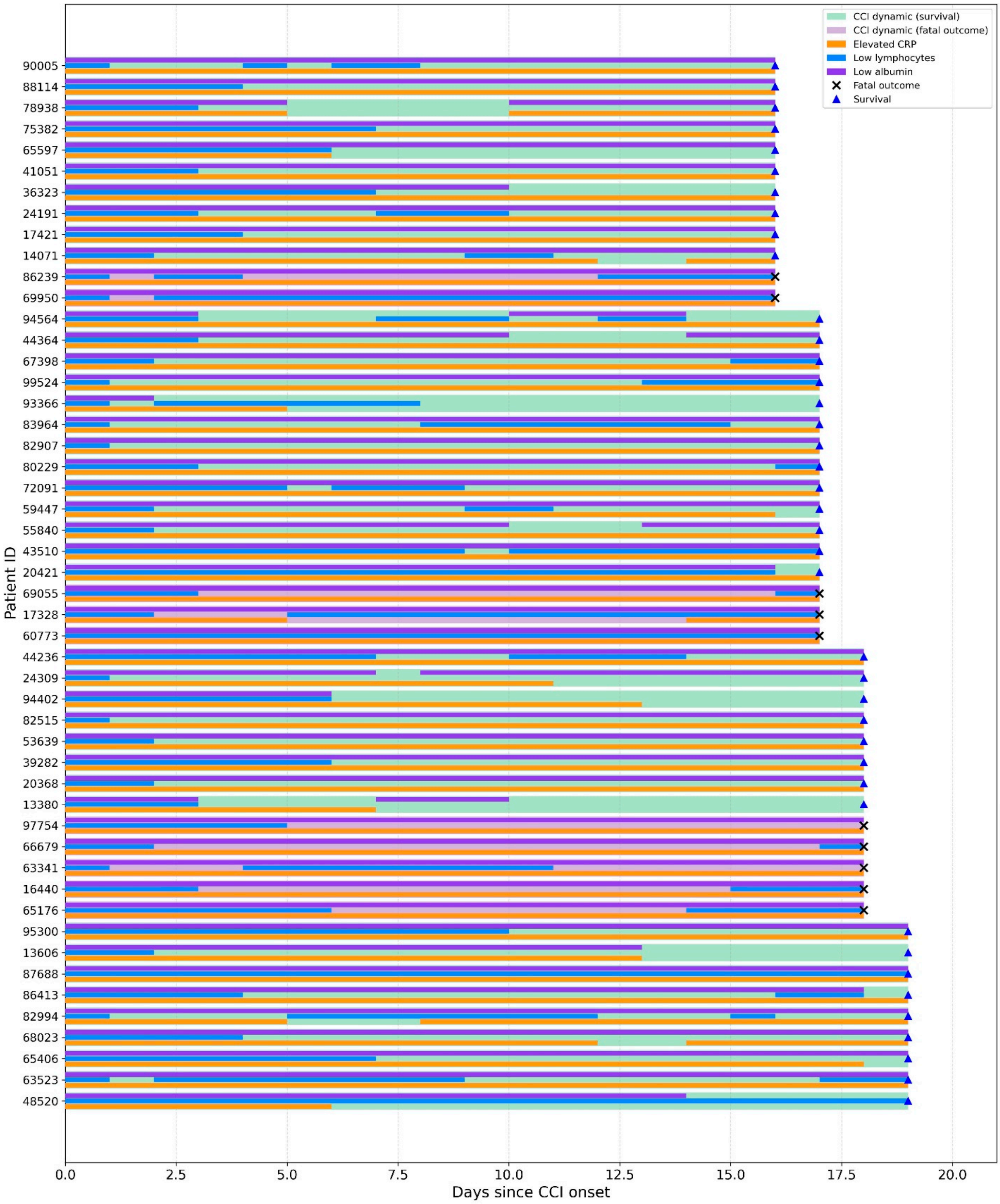

Supplementary Figure S9. Swimmer plot of CCI-related biomarker domain trajectories after CCI onset (part 9).

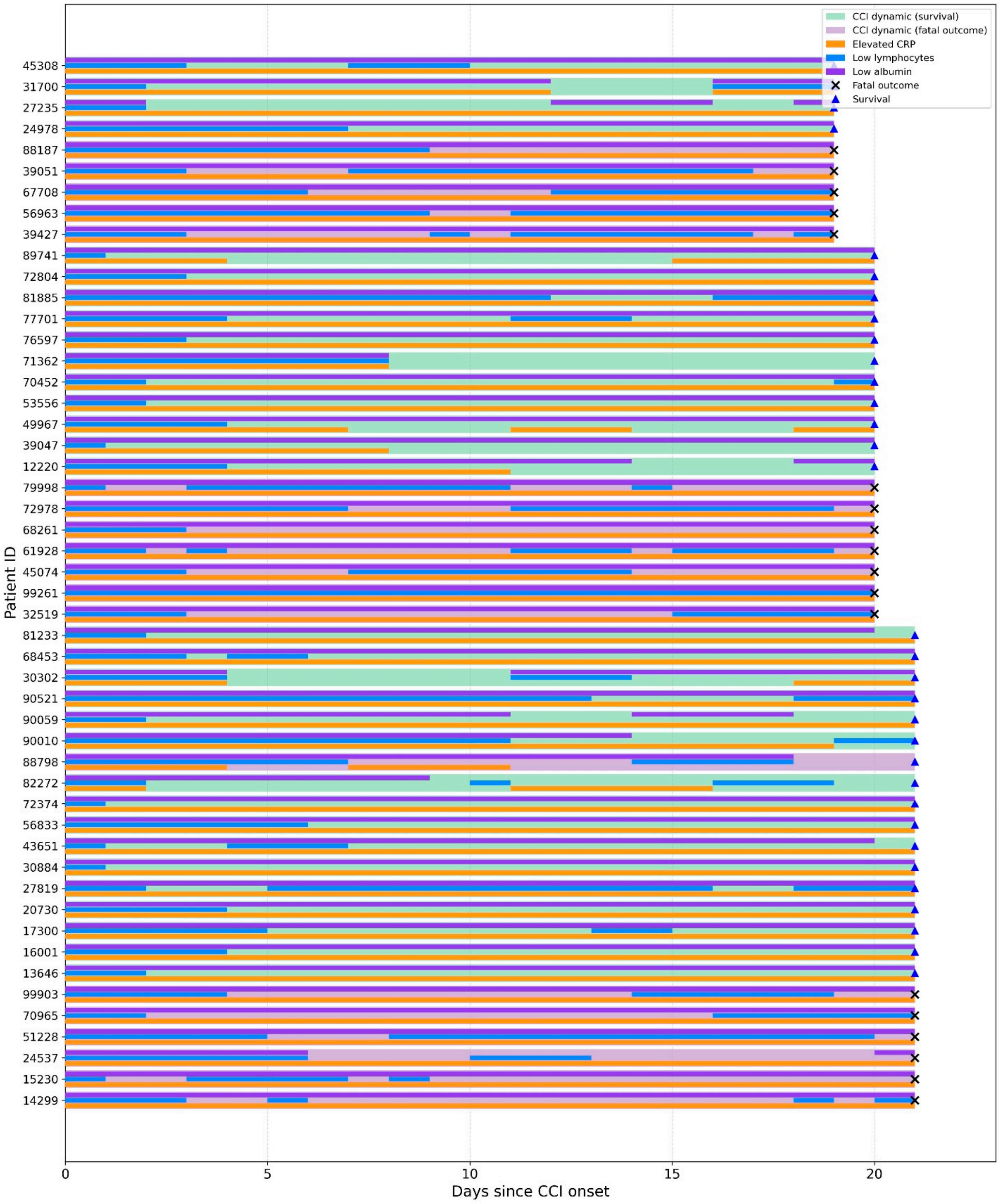

Supplementary Figure S10. Swimmer plot of CCI-related biomarker domain trajectories after CCI onset (part 10).

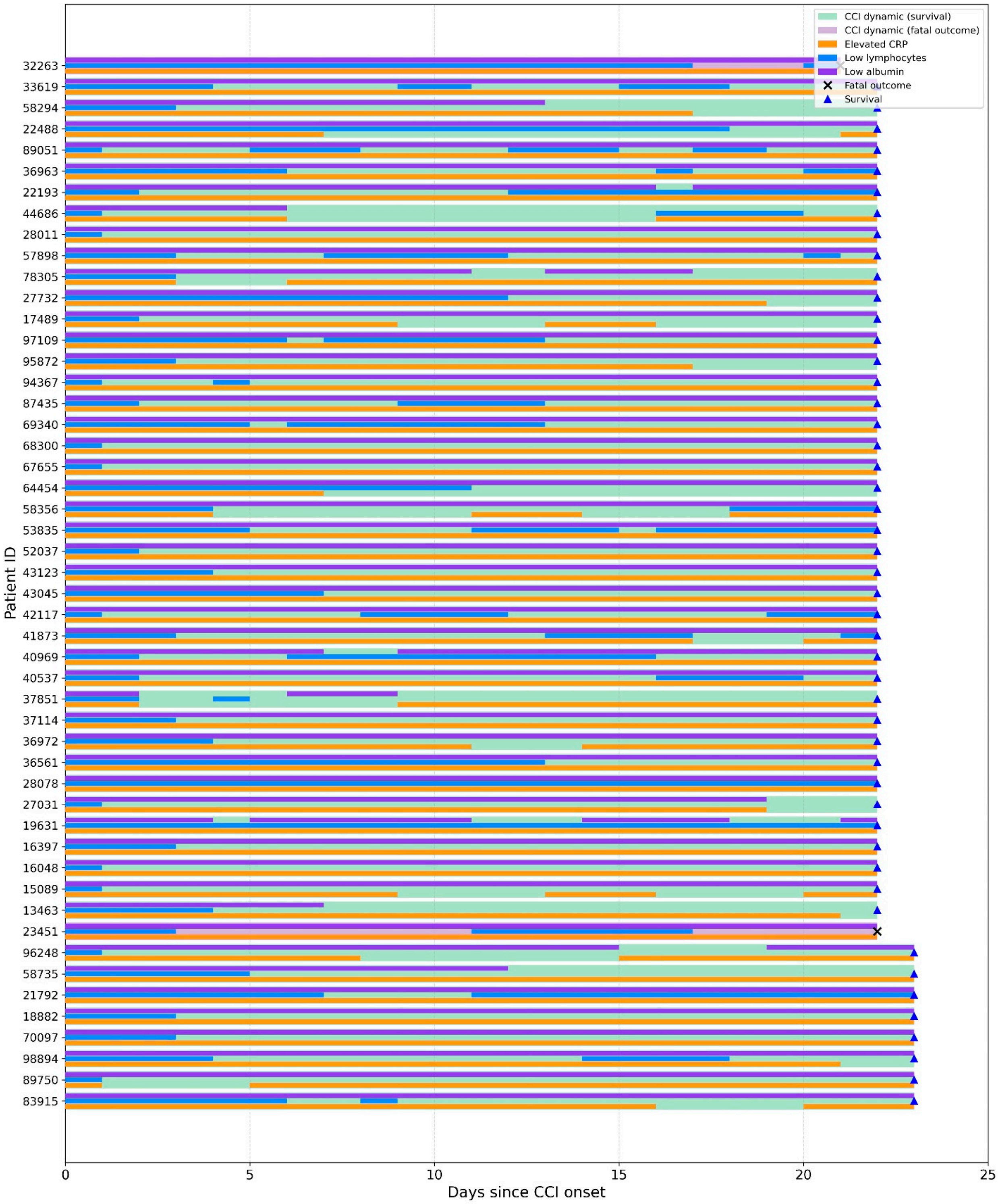

Supplementary Figure S11. Swimmer plot of CCI-related biomarker domain trajectories after CCI onset (part 11).

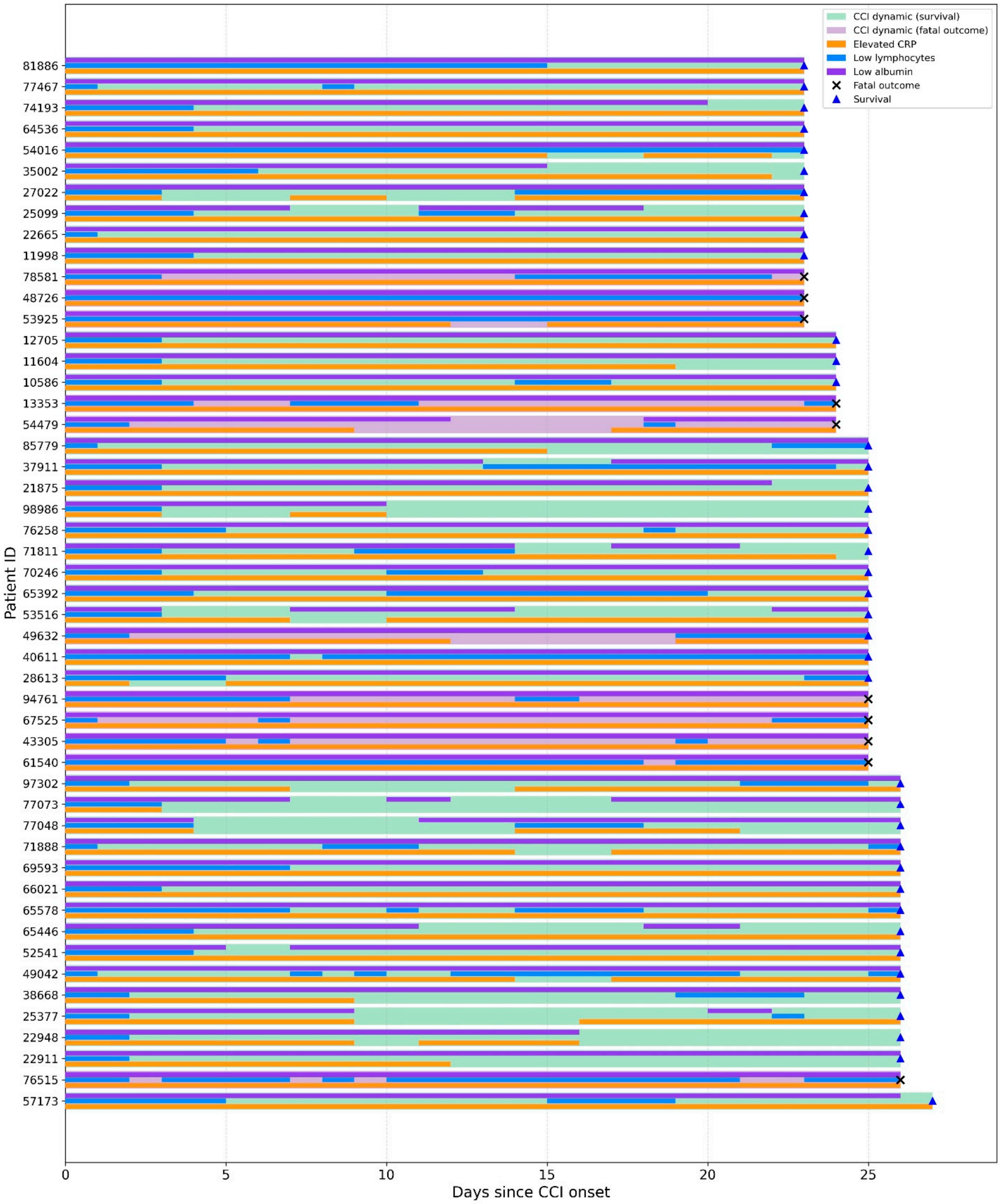

Supplementary Figure S12. Swimmer plot of CCI-related biomarker domain trajectories after CCI onset (part 12).

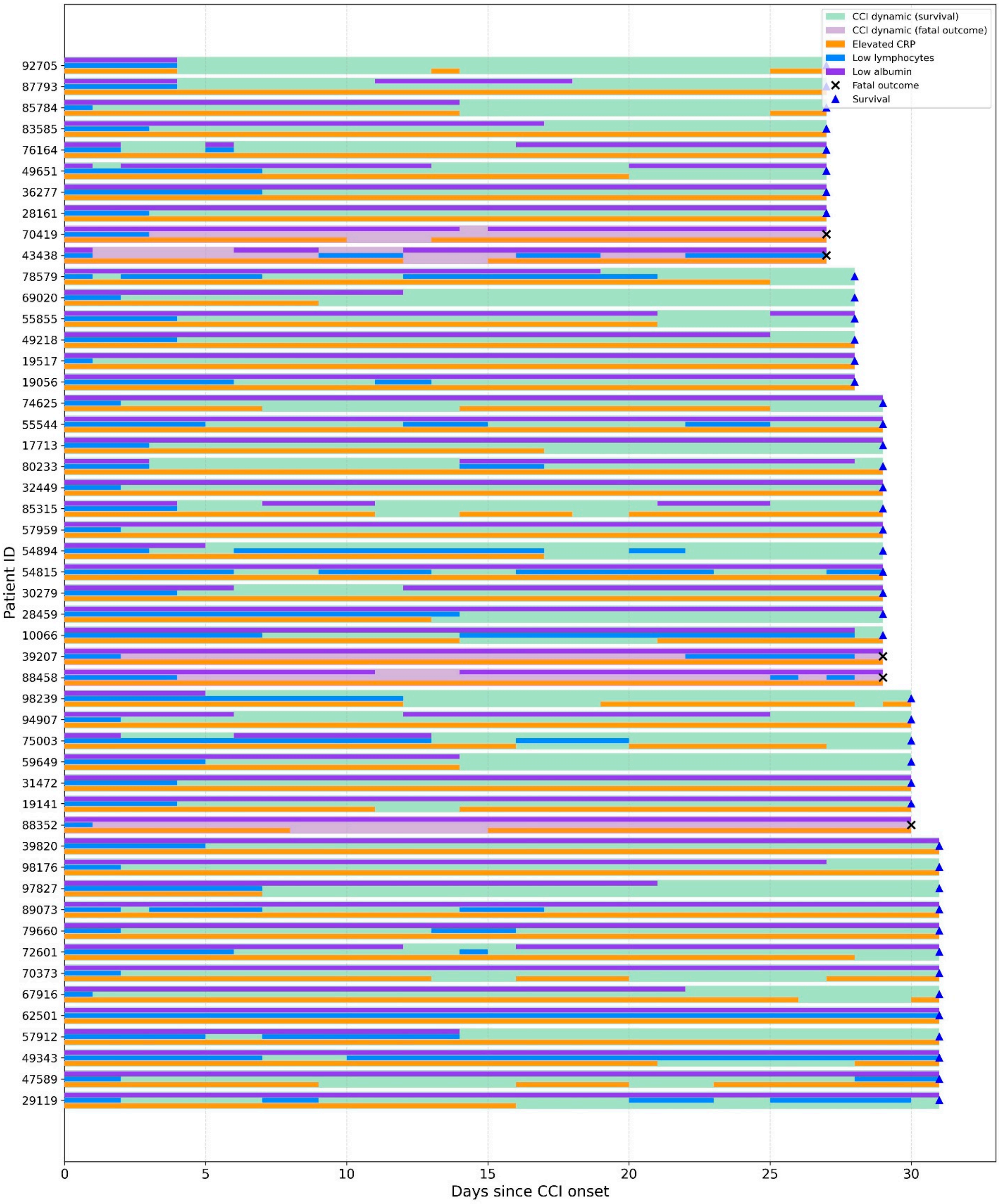

Supplementary Figure S13. Swimmer plot of CCI-related biomarker domain trajectories after CCI onset (part 13).

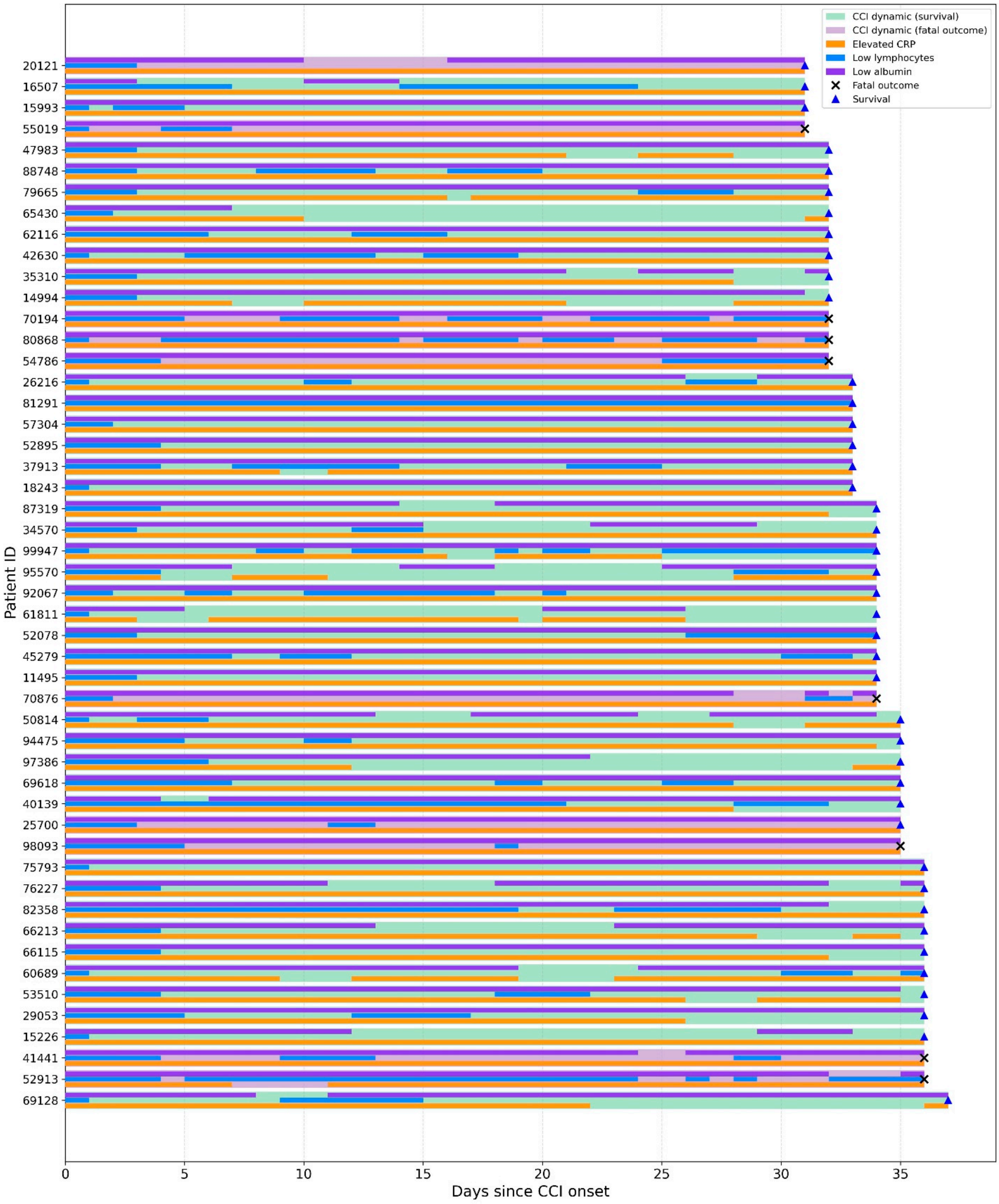

Supplementary Figure S14. Swimmer plot of CCI-related biomarker domain trajectories after CCI onset (part 14).

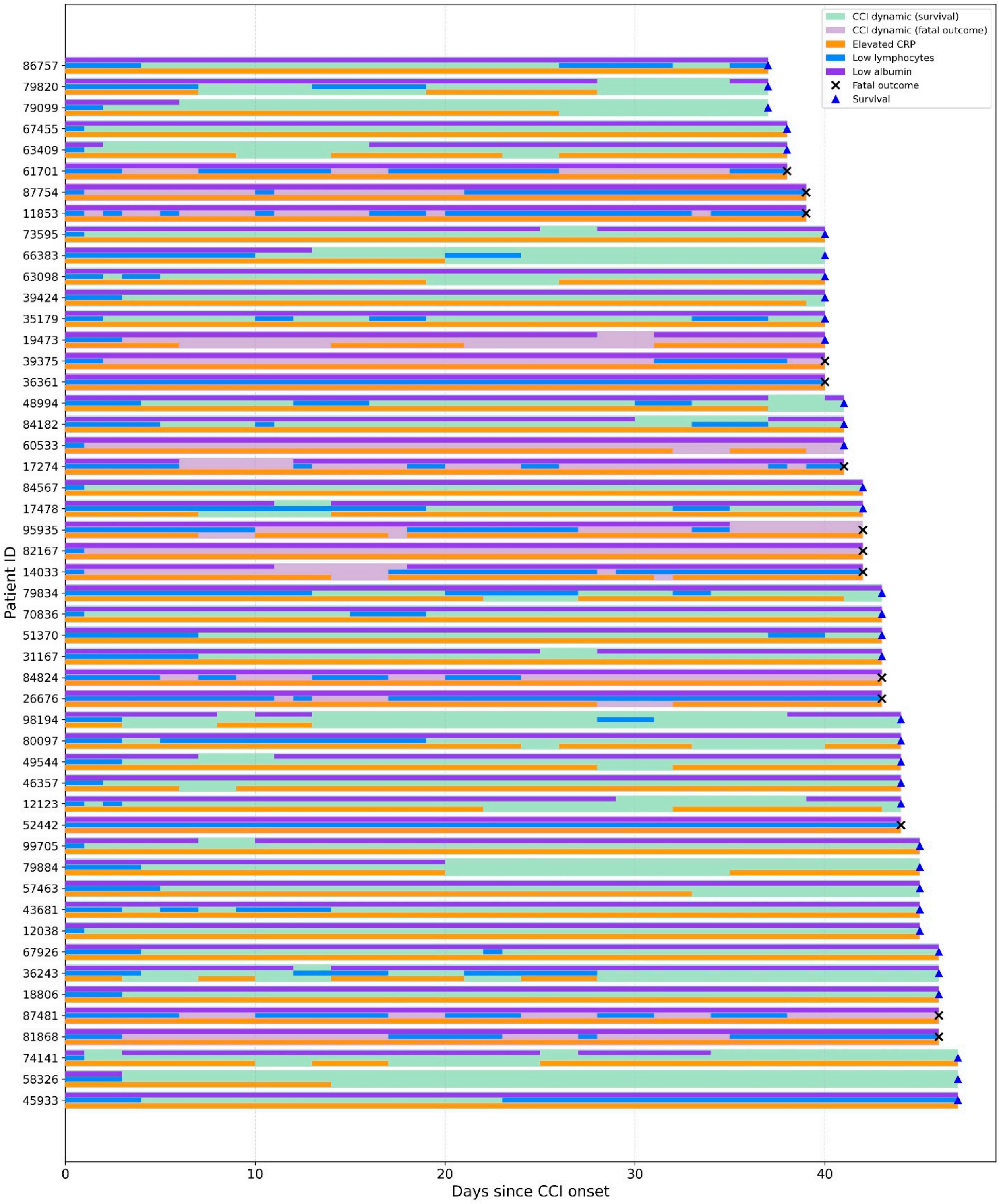

Supplementary Figure S15. Swimmer plot of CCI-related biomarker domain trajectories after CCI onset (part 15).

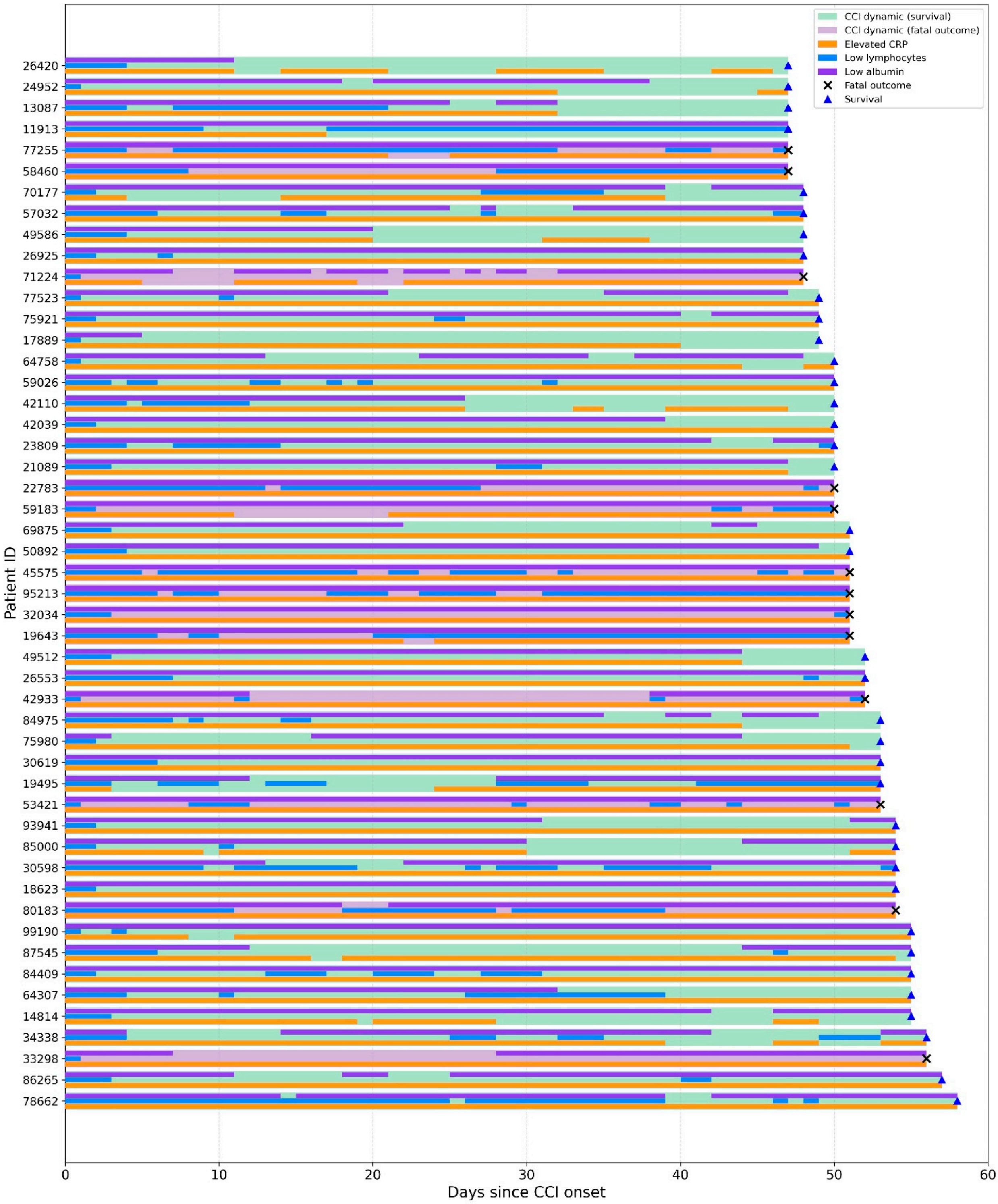

Supplementary Figure S16. Swimmer plot of CCI-related biomarker domain trajectories after CCI onset (part 16).

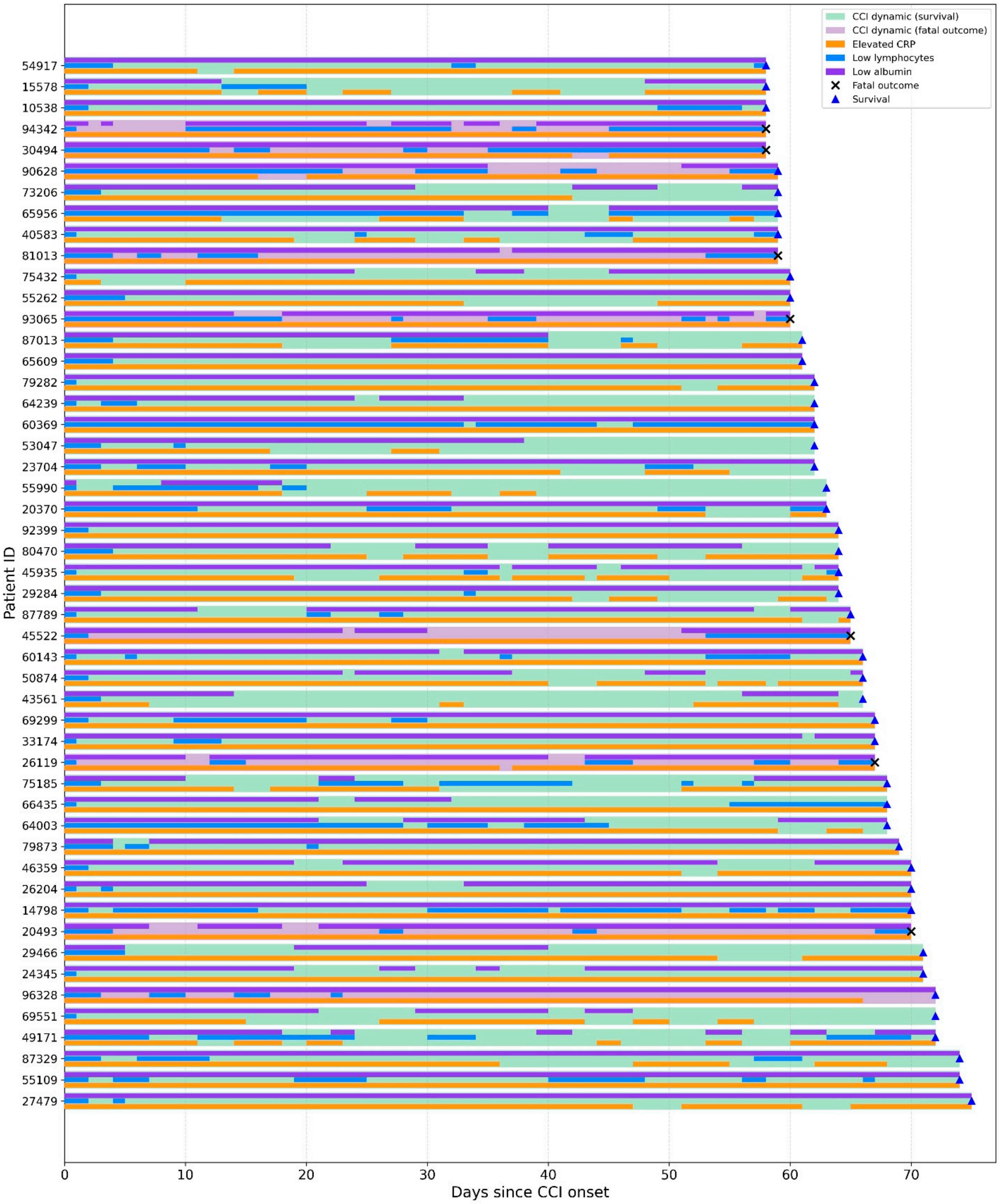

Supplementary Figure S17. Swimmer plot of CCI-related biomarker domain trajectories after CCI onset (part 17).

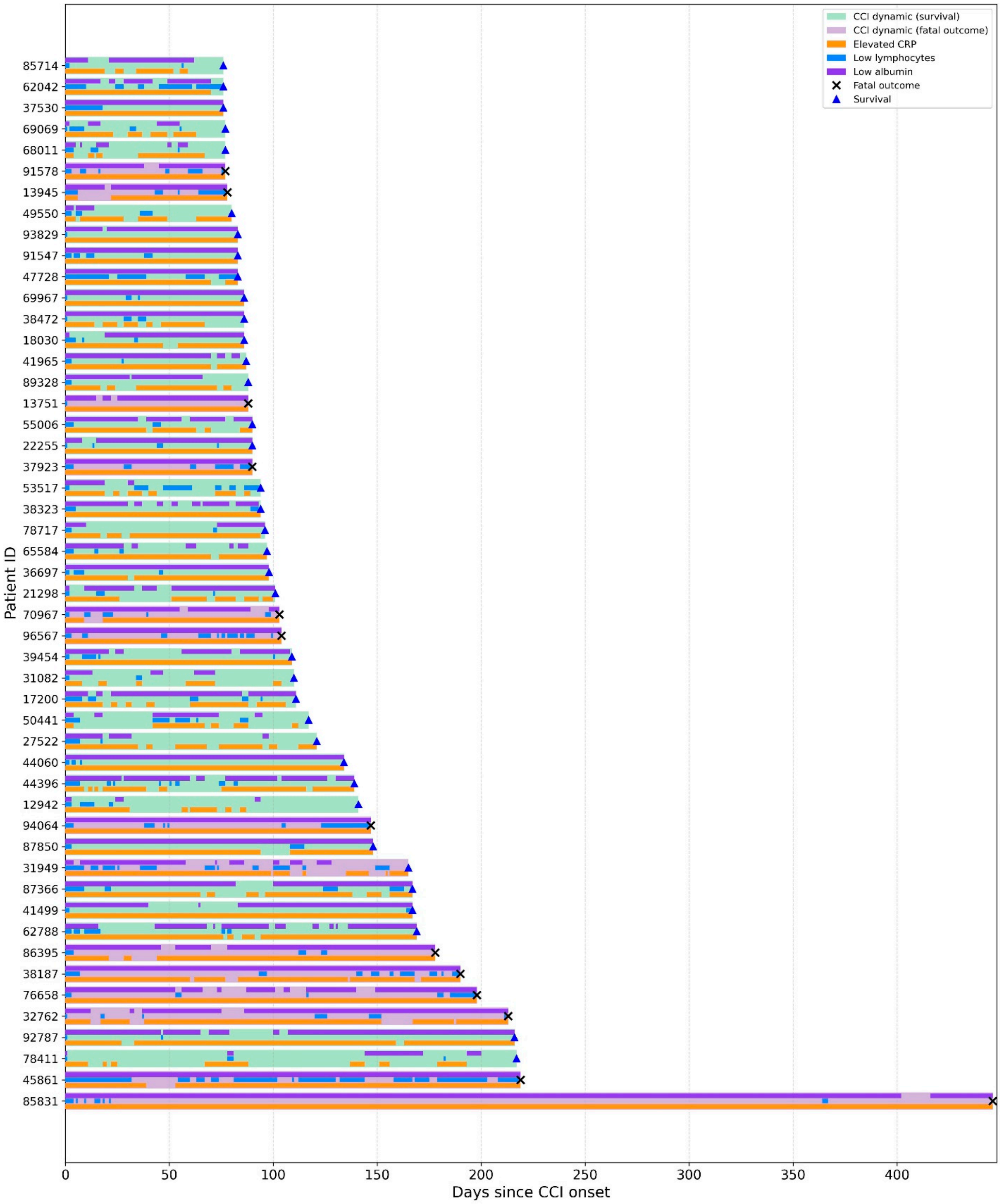

Supplement: Supplementary file 1 [file medicina-62-01686-s001.zip › medicina-4485877-supplementary.pdf]
